# Multifaceted Janus Textile Simultaneously Achieving Self-Sustainable Thermal Management, Perception, and Protection

**DOI:** 10.1007/s40820-025-02030-6

**Published:** 2026-01-13

**Authors:** Jialong Chai, Guilong Wang, Runze Shao, Lin Ni, Guoqun Zhao, Jintu Fan

**Affiliations:** 1 State Key Laboratory of Advanced Equipment and Technology for Metal Forming, Shandong University, Jinan, 250061 Shandong People’s Republic of China; 2 Key Laboratory for Liquid-Solid Structural Evolution and Processing of Materials (Ministry of Education), Shandong University, Jinan, 250061 Shandong People’s Republic of China; 3 School of Fashion and Textiles, The Hong Kong Polytechnic University, Hung Hom, Kowloon, Hong Kong, 999077 People’s Republic of China

**Keywords:** Multifunctional textiles, Janus structure, Personal thermal management, Self-powered sensing, Protective textile

## Abstract

The integration of personal thermal management, perception, protection, and comfort is essential for the development for next-generation textiles capable of performing in complex environments. Janus-designed textiles represent a promising route to this integration, offering adaptive dual-functionality. However, most current designs are limited to single-purpose applications, restricting their effectiveness in truly multifunctional scenarios. Here, we present a multifaceted Janus (X-Janus) textile that overcomes these limitations by combining innovative microporous polytetrafluoroethylene fibers with multidimensional nano- to microscale fibrils and MXene-coated carbon fabric. The X-Janus textile delivers multiple energy-independent functionalities: a spectral Janus design that enables adaptive thermal management through switchable radiative cooling or warming; an electrical Janus design that provides self-powered sensing and energy harvesting; and a wetting Janus design that ensures wearing comfort with waterproofness. Besides, the textile provides comprehensive protection including chemical resistance, electromagnetic interference shielding (56 dB), ultraviolet protection (UPF > 1,500), and flame retardancy. By integrating these advanced features, the X-Janus textile inspires new strategy for self-sustainable textiles, offering scalable solutions for outdoor safety, industrial wearables, and intelligent clothing where multifunctionality and environmental resilience are critical.

**Supplementary Information:**

The online version contains supplementary material available at 10.1007/s40820-025-02030-6.

## Highlights

- A shear-induced fibrillation strategy enables the continuous fabrication of microporous polytetrafluoroethylene fibers with nano-micro-fibrils with great porosity and strength.
- A multifaceted Janus design integrates spectral, electrical, and wetting dualities in one textile, realizing adaptive cooling/heating, self-powered sensing, and waterproof breathability without external energy.
- Comprehensive protection including electromagnetic interference shielding, UV resistance, flame retardancy, and chemical stability for self-sustainable, multifunctional, and comfortable wearables.

**Supplementary Information:**

The online version contains supplementary material available at 10.1007/s40820-025-02030-6.

## Graphical abstract

**Figure:**
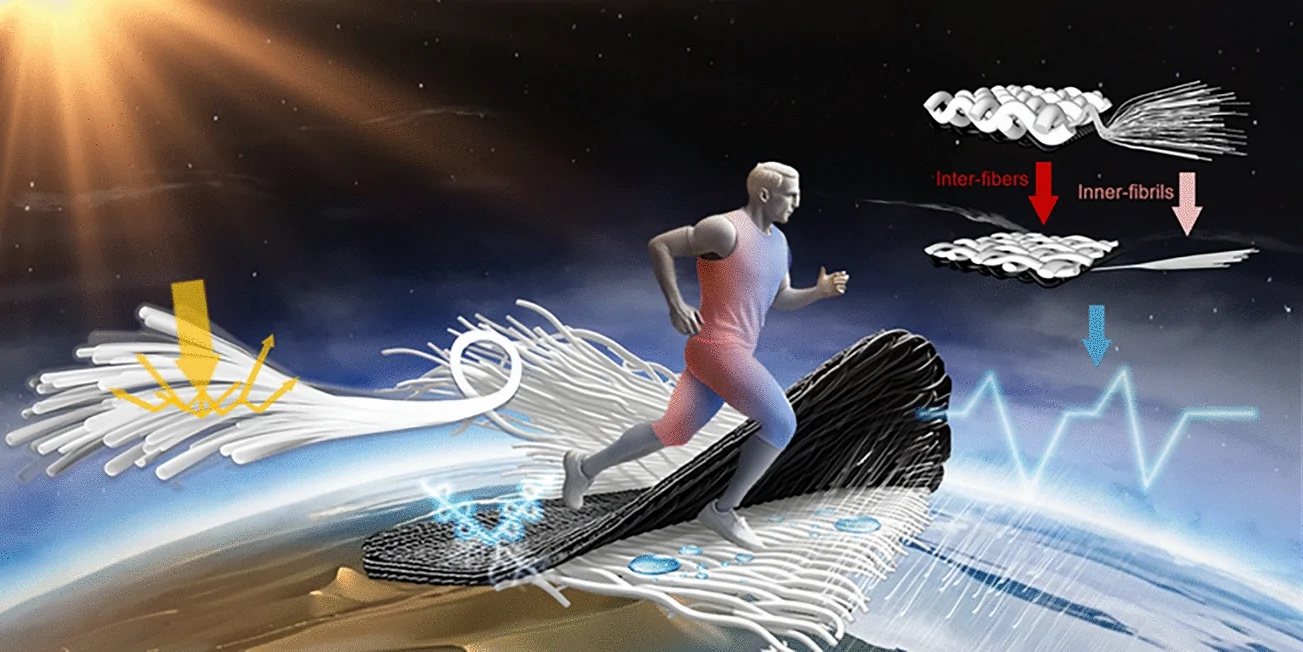

## Introduction

**Fig. 1:**
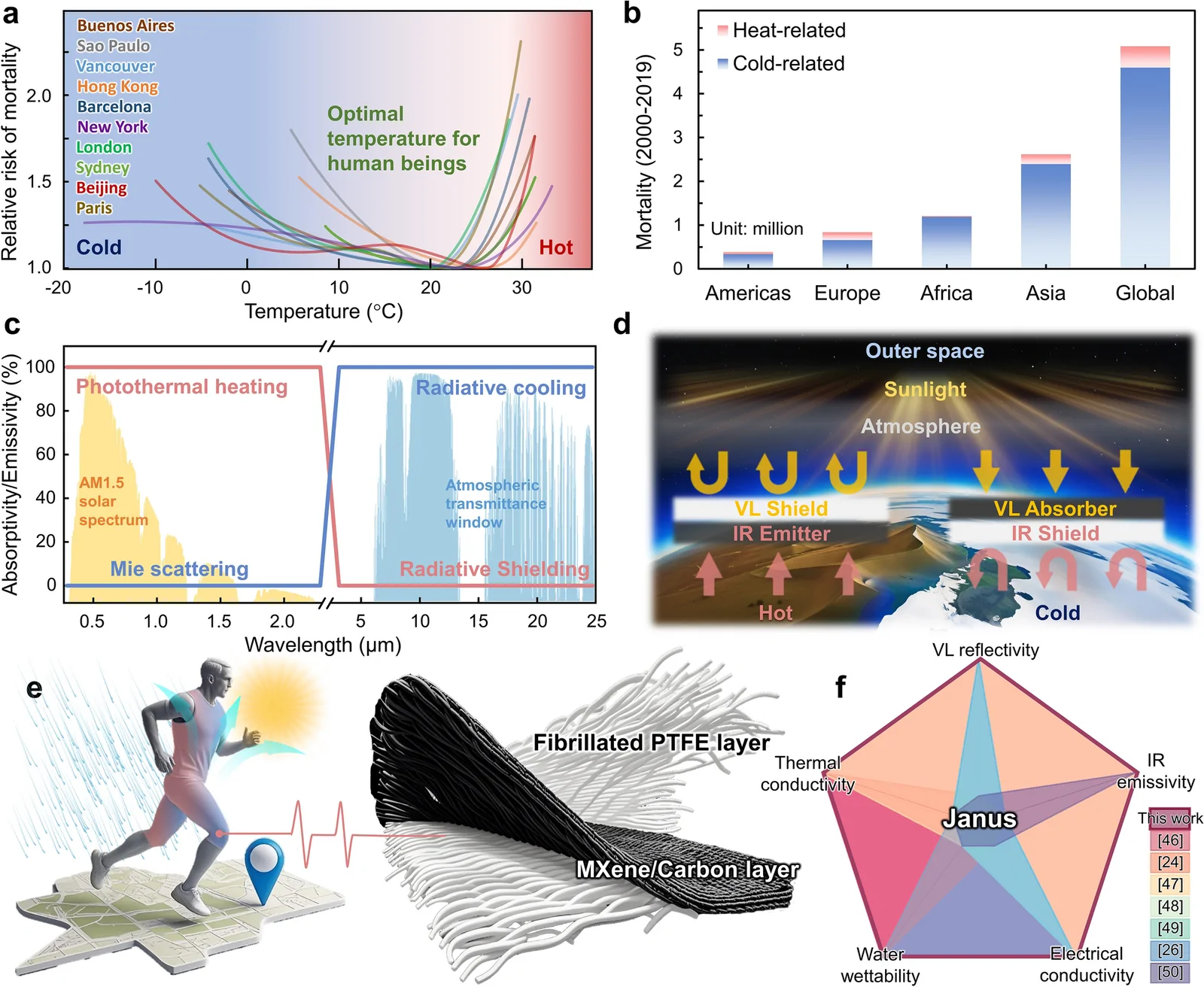
Design of multifaceted Janus textile. **a** Relationship between mortality risk and environment temperatures in different regions from Ref. [1], Springer Nature Ltd.** b** Statistic of temperature-related mortality in 2000–2019 from Ref. [2], Elsevier Sci. Ltd. **c** Ideal spectral Janus design for effective radiative PTM textiles. **d** Schematic of the Janus textile with radiative PTM function by switching in hot and cold environments. **e** Schematic of the design of multifaceted Janus textile (left) with PTM, perception, and protection functions (right). **f** Comparison of multifaceted Janus properties between this work and previously reported textiles [24, 26, 46–50]

Global climate change is rapidly transforming human living environments, with increasingly frequent extreme weather events leading to severe temperature-related illnesses that endanger personal safety, particularly for manual workers in harsh conditions (Fig. 1a) [1]. For outdoor workers and extreme sports enthusiasts, drastic temperature fluctuations and complex environments pose life-threatening risks due to failures in thermal regulation (Fig. 1b) [2]. There are already numerous examples where sudden weather changes result in body temperature imbalance, highlighting critical flaws in conventional protective gear's ability to integrate personal thermal management (PTM), intelligent perception, and hazard protection [3]. Traditional thermal management systems, especially for protective clothes, achieve temperature regulation through embedded electronic elements that heavily rely on external power supplies [4–6], resulting in increased weight and excessive energy consumption [7]. More critically, existing protective textiles (e.g., military, firefighter, space, and biohazard suits) adopt independent designs for thermal, electronic, and protective layers [8–12], which not only increases manufacturing costs but also results in thicker textiles with reduced wearing comfort. Therefore, overcoming these multifaceted threats demands advanced fabrics that simultaneously unify thermal regulation, intelligent perception, and diverse protection. However, the limitation of conventional textiles lies in the trade-off between “energy-function-comfort trilemma”. Developing advanced textiles that achieve multifunctionality and comfort synergy through structural design that without external energy supply becomes pivotal to overcoming these bottlenecks.

To address the energy trilemma in textiles, passive thermal management technologies have attracted wide attention (e.g., radiative cooling/heating [13–16], phase change textiles [17–20]. However, existing solutions remain functionally isolated and lack adaptability across dynamic environments. The Janus design, characterized by intrinsic dual-sided heterogeneity, has been used in advanced textiles as a promising platform [21–25]. Parallelly constructed, non-interfering functional modules on both sides of a textile, achieved through material property differences, can overcome the “single-scenario applicability” limitation. For instance, spectral Janus textiles leveraging optical property differences enable effective radiative thermal management [26–30], adapting to diurnal or year-round temperature variations without energy input. Electrical Janus materials serve as fundamental components for electric circuits, enabling sensor integration [31], power generation [32], electromagnetic shielding [33], and soft robotics [22]. Chemical heterogeneity at the Janus interface (e.g., hydrophilic/hydrophobic surfaces) induces special wetting properties, enabling waterproofness with breathability [34, 35] or directional liquid transport for moisture management [36–38]. For instance, Zhang et al. constructed a bilayer Janus textile with a hierarchical gradient structure that enabled directional water transport, passive radiative cooling, and smart sensing capabilities [24]. Hsu et al. developed a dual-mode PTM textile composed of coupled carbon and metal layers, exhibiting distinct infrared emissivity on each side and thereby achieving effective thermal comfort through bidirectional thermal regulation [26]. These pioneering studies have demonstrated the potential of asymmetric textile architectures to achieve environment-responsive functionalities. However, the functionality of conventional Janus structures remains largely limited to isolated or single-domain properties. Most reported systems focus on a specific aspect such as thermal regulation, moisture management, or sensing without achieving coordinated performance among multiple functions. This limitation prevents them from satisfying the increasingly stringent requirements for next-generation advanced textiles, which demand simultaneous adaptability, comfort, intelligence, and protection under complex or dynamic conditions.

This study aims to develop a multifaceted Janus (X-Janus) textile that achieves synergistic integration of diverse functions, thereby producing a monolithic textile with versatile characteristics to meet comprehensive wearing requirements. We pioneer a shear-induced fibrillation strategy to fabricate continuous microporous polytetrafluoroethylene (PTFE) fibers for woven textiles. The microstructure is engineered into unidirectional and multidimensional fibrils ranging from the nano- to microscale, enabling efficient broadband solar scattering. By coupling these fibers with MXene-coated carbon fabric, the resulting spectral and thermal Janus features enable adaptive passive PTM applications in both hot and cold environments. The electrical Janus feature provides a significant triboelectric effect, offering sensing and powering functions in various scenarios. The wetting Janus feature ensures excellent breathability and high waterproofness, thereby enhancing wearing comfort and providing exceptional protection against diverse hazardous impacts, including superior flame retardancy. Notably, the integration of efficient thermal management, perception, and protective functionalities with versatile environmental adaptability does not require external energy supply, significantly enhancing the self-sustainability of intelligent textiles.

## Experimental Section

### Materials

PTFE powder (METABLEN-A3800) was purchased from Mitsubishi Rayon Chemical, Japan. Polylactic acid (PLA, Ingeo4032D) was purchased from NatureWorks LLC, USA. The Ti_3_AlC_2_ (400 mesh) and Celgard 3501 membrane (50 mm) was provided by Jilin Province 11 Technology Co., Ltd. Hydrochloric acid (HCl, 37 wt%), lithium fluoride (LiF),* n*-butyl alcohol, and dichloromethane (DCM) were purchased from Aladdin Biochemical Technology Co., Ltd., Shanghai. Carbon fabric (W0S1011) was purchased from CeTech, China.

### Preparation of Multifaceted Janus (X-Janus) Textiles

The raw materials of PTFE and PLA were fed into a twin-screw extruder (SJZS-10B, Ruiming, China) at a preset processing temperature. The resulting melt mixture was extruded through a die and subsequently collected as continuous PTFE/PLA composite fibers via traction winding. The as-prepared composite fibers were repeatedly washed with dichloromethane and then subjected to Soxhlet extraction at 60 °C to fully remove the PLA. After drying, microporous PTFE fibers were obtained and subsequently woven into textiles using a plain weave pattern, with the fibers arranged as both warp and weft by hand knitting. To prepare the MXene-coated carbon fabric, MXene dispersion was synthesized using a minimal delamination method (Note S1) [33], and it was then deposited onto acid-treated carbon fabric by spray coating and drying.

### Characterization

#### Structural and Mechanical Characterizations

The microstructures of the prepared fibers were examined using a thermal field scanning electron microscope (SEM, SU-70, Hitachi, Japan). The diameter distribution of PTFE fibrils was quantitatively measured from the SEM images using Image-Pro Plus software, with at least 100 fibrils analyzed for each sample. The porosity was determined by a gravimetric method [6], in which the amount of liquid retained in the fiber was measured using *n*-butyl alcohol as the wetting liquid. Wide-angle X-ray diffraction (WAXD) was performed using an X-ray diffractometer (HomeLab, Rigaku, Japan). The tensile properties of the microporous PTFE fibers were evaluated with a universal testing machine (HDW-2000, Hengxu, China) equipped with a specialized fiber fixture.

#### Spectral and Thermal Measurements

The UV–Vis–near-IR spectra (0.3–2.5 μm) were measured using an ultraviolet–visible–near-infrared spectrophotometer (Cary 7000, Agilent, USA). The mid-infrared spectra (2.5–25 μm) were obtained using a Fourier transform infrared (FTIR) spectrometer (Nicolet iS50, Thermo Scientific, USA) equipped with a gold integrating sphere. The infrared transmission of microporous PTFE fibers was measured using a spectrometer (Nicolet 6700/NXR, Thermo Fisher, USA) with an ATR module. Thermal conductivity was measured using a hot disk transient plane source (TPS 2500S, Hot Disk) method. Circular samples (diameter = 25 mm, thickness = 0.2–0.5 mm) were sandwiched around a Kapton-insulated sensor. The tests were conducted in Jinan, China (36.65°N, 117.12°E), the cooling test was performed on a summer clear day, and a warming test was performed on a winter clear day. Human skin was simulated by a silicone-encapsulated heater connected to a direct current power source, providing approximately 140 W m^−2^ heating power to mimic body heat. Thermal conductivities were measured using a thermal constant analyzer (TPS 2500 S, Hot Disk, Sweden). The scattering efficiency was calculated using finite-difference time-domain (FDTD) simulations. Theoretical cooling and heating powers were calculated using Matlab software (Note S2).

#### Electrical and Triboelectric Measurements

The surface resistivity of the textile was measured using an electrometer (Model 6517B, Keithley, USA) equipped with a resistivity test fixture (Model 8009). To evaluate the perception abilities, real-time triboelectric output signals were collected by connecting the MXene/carbon layer to the programmable electrometer and monitored using a LabVIEW program on a computer. A linear motor was used to provide cyclical contact–separation motions for long-term testing. The electric generation tests were conducted by connecting the textile to different capacitors and measuring the resulting voltage.

#### Comprehensive Wearing and Protection Tests

The water contact behaviors were observed by depositing a small water droplet (5 μL) onto the PTFE fiber and MXene-coated carbon fabric, respectively, using a contact angle tester (JC2000D, Powereach, China), with the process recorded by a high-speed camera. The water vapor transmission rate (WVT, g m^−2^ (24 h)^−1^) was measured and calculated according to GB/T 12704.2–2009. The electromagnetic interference (EMI) shielding effectiveness (SE) was measured using a vector network analyzer (N5247A, Agilent, USA) in the frequency range of 8.2–12.4 GHz (X-band), with textile samples sized 22.86 × 10.16 mm^2^ (Note S3). The UV-protective performance was evaluated using a UV–Vis–NIR spectrophotometer (Lambda 950, PerkinElmer, USA), and the UPF value was calculated according to GBT18830-2009. Chemical resistance was assessed by immersing the textile in strong acid (H_2_SO_4_, 98%) and alkaline (NaOH, 60%) solutions for 30 min. Flame retardancy was evaluated by vertically placing the textile above a flame for 10 s.

## Results and Discussion

### Design of Multifaceted Janus Textile

The principle for designing multifaceted Janus textile (name as X-Janus textile) is to integrate multi-functionalities with contrasting features into single textile. Despite the various reported single-functional Janus textile with diverse properties, there still lacking suitable materials that simultaneously satisfy the desired spectral, electric, and wetting properties. For efficient PTM function, both the spectral and thermal properties should be considered to regulate the thermal transfer through radiative and conductive routes. From spectral perspective, the Janus PTM textiles should have distinct visible light (VL) absorption and infrared (IR) reflection behaviors on each side, which requires specially designed textile materials with specific nano/micro structures. The ideal spectral design is shown in Fig. 1c, for the cooling purpose in hot environments, a low VL absorption with high IR emission across a wide infrared range facilitate to effectively shield the solar radiation while dissipate the heat into surroundings and outer space through atmospheric window (Fig. 1d). Conversely, for warming purpose in cold environment, the textile should possess high VL absorptivity for photothermal conversion while high IR reflectivity (low absorptivity) to retain the body heat by shielding the strong IR radiation from human skin. Meanwhile, for contact thermal comfort, it should be thermal insulating in cold environment but thermal conductive in hot environment.

To satisfy the requirement for strong Mie scattering at the range of solar light and high IR emittance at atmosphere window (8–13 μm) [15, 39–41], we employ the microporous PTFE fibers woven textile as the effective passive cooling layer because of its high VL reflectivity and IR transparency. Meanwhile, the Mxene-coated carbon fabric was selected as the warming layer (Fig. 1e). For one, carbon fabric shows high IR emissivity at broad spectral range, superior to human skin and most of the polymer-based materials [42]. For another, the coupling of MXene coating enables perfect photothermal conversion property at the warming side, while the high IR reflection can greatly prevent the human heat loss [43]. Besides, PTFE is one of the most ideal triboelectrical negative materials that shows exceptional electro affinity. The combination of conductive carbon/MXene layer perfect aligns with the design principle for single-electrode type triboelectrical sensor and generator [44, 45]. This electrical Janus design enables great contact electrification when being subjected to external stimuli, unlocking more self-powered intelligent functions. Thereby, the designed textile can not only enable efficient PTM in both cold and hot environment by switch the corresponding side of the clothes, but also capable of electrical output to support smart perception and energy supply for electric devices. Moreover, for desired wearing comfort, the hydrophobic PTFE layer and hydrophilic carbon/MXene layer achieve suitable wettability, and a woven structure is required to ensure great air permeability, which is of vital importance to maintains breathability for body moisture management with effectively proofness to external impact such as rain droplet and strong wind, ensuring adequate wearable comfort for human beings and adapting various environments during practical applications. Therefore, as shown in Fig. 1f, the newly developed X-Janus textile integrates multiple asymmetric features in spectral, electrical, thermal, and wettability differences, achieving superior multifunctional performance and wearability compared with previously reported Janus textiles [24, 26, 46–50].

### Processing and Characterization of Microporous PTFE Fibers

**Fig. 2:**
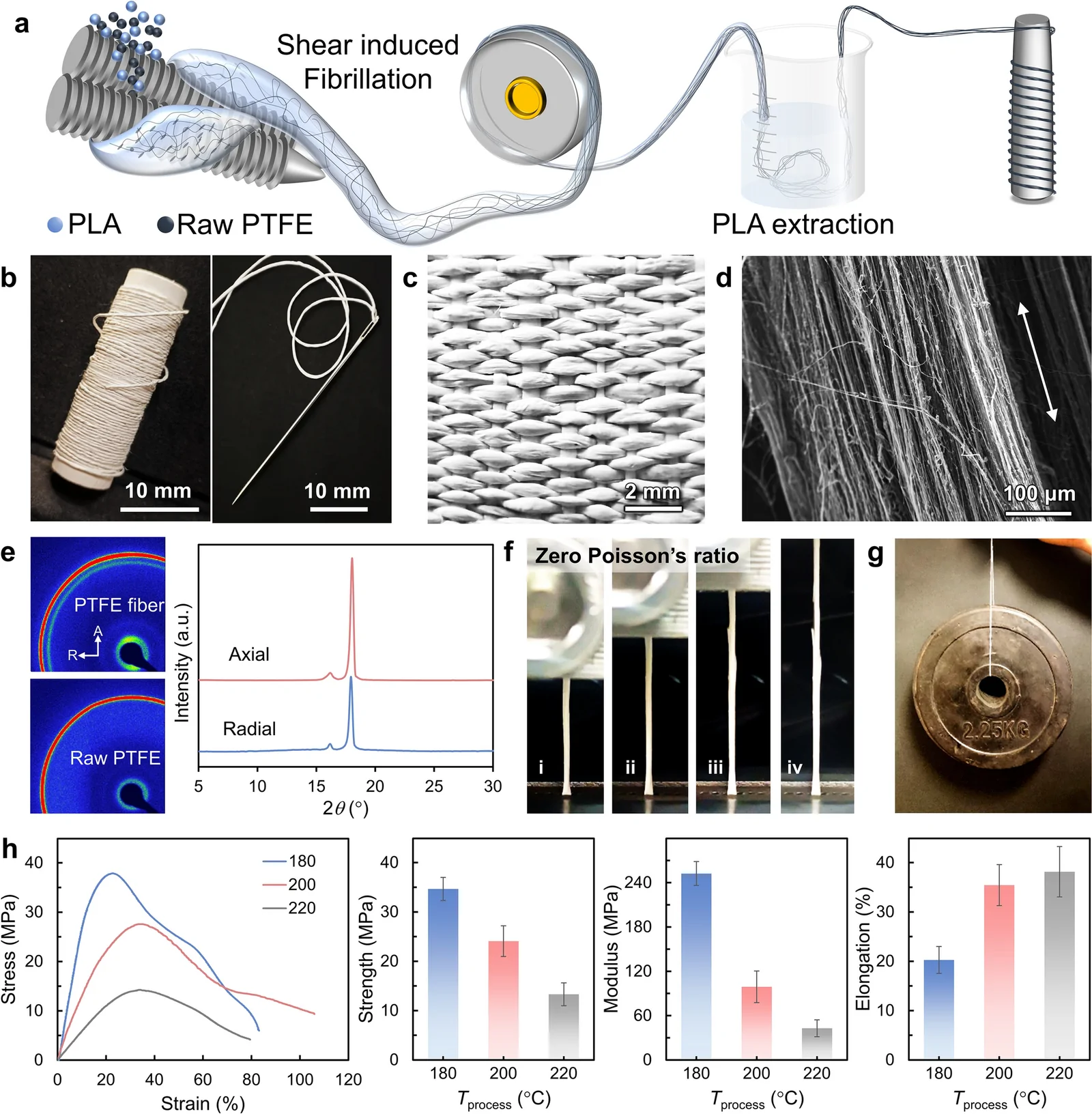
Fabrication and characterization of microporous PTFE fiber. **a** Schematic of the in-situ fibrillation methodology for processing fibers. **b** Photograph showing a microporous PTFE fiber collected in a roll (left) and threaded in a needle (right). **c** Photograph showing the PTFE fiber woven fabric using a classic plain weaving pattern. **d** SEM image of the fiber microstructure with unidirectional fibrous morphology. **e** 2D-WAXD pattern of PTFE fiber and raw PTFE, respectively (left), and 1D-WAXD profile of the PTFE fiber along the axial and radial directions (right).** f** Tensile stretching process showing the zero-Poisson’s ratio behavior of PTFE fiber. **g** Photograph of a single fiber supporting a weight of 2.25 kg, demonstrating its great tensile strength. **h** Tensile tests of the microporous PTFE fibers produced at different *T*_process_ (180, 200, 220 °C) including strain–stress curves, strength, modulus, and elongation (the elongation is determined by the strain at the maximum stress due to the unique tensile behavior of PTFE fiber)

Although PTFE has been widely used as a waterproof and breathable membrane in textiles for decades due to its excellent hydrophobicity, thermal stability, and chemical resistance, there is still no versatile approach for producing microporous PTFE fibers [6, 15]. This remains the greatest challenge in achieving the designed X-Janus textile. To meet the practical large-scale demands of the textile industry, we first developed continuous microporous PTFE fibers using an innovative shear-induced fibrillation spinning methodology [51–53]. As illustrated in Fig. 2a, raw PTFE, together with bio-sourced PLA as a processing agent, was compounded using a universal twin-screw extruder at temperatures above the melting point of PLA. Under strong shear force originates from the meshing rotation of the twin screws and their synergistic effect of shearing and conveying, the PTFE crystals spontaneously unwind into nanofibrils. PTFE itself has a linear helical chain structure that enables the slippage between the crystals, allowing the originally curled helical chains to stretch and unwind into discrete nanofibrils with a diameter usually ranging from tens to hundreds of nanometers. These nanofibrils then interweave to form an entangled nanofibril network. The highly viscous melt is then extruded through the die of the twin-screw extruder and subjected to traction spinning, resulting in continuous and uniform composite fibers. Figure S1 shows photographs of the extrusion and spinning process using an industrial-scale extruder, and Fig. S2 presents the obtained composite fibers and the nanofibrils embedded in PLA, confirming the formation of the PTFE fibril network. Subsequently, the microporous PTFE fiber can be obtained by extracting most of the PLA phase by solvent washing and ambient drying (Fig. S3), while the skin layer remains relatively solid texture that avails protective application (Fig. S4). As shown in Fig. 2b, spools of microporous PTFE fibers exhibiting a fully milky white color and excellent softness can be produced and readily knitted into woven structures (Fig. 2c), enabling great wearing comfort.

Notably, as presented in Fig. 2d, the microporous fiber displays an oriented fibrous structure along the axial direction, achieved by directional traction-induced alignment of the nanofibril network. The oriented nanofibrils also result in directional crystals; as shown in Fig. 2e, the PTFE fiber exhibits an anisotropic pattern in the two-dimensional X-ray scattering image, with stronger signals along the axial direction, whereas the raw PTFE shows a homogeneous pattern. This unique feature significantly influences the mechanical properties of the fiber. As shown in Fig. 2f, the fiber macroscopically demonstrates a unique zero-Poisson’s ratio behavior when subjected to tensile stretching along the axial direction, as the stress is distributed among the isolated nanofibrils. Therefore, by preventing the radially propagation of cracks, such an oriented structure can provide superior strength along the axial direction (Fig. S5). In Fig. 2g, a single fiber with a diameter of about 500 μm can lift a weight of 2.25 kg, more than 10,000 times its own weight. However, the mechanical strength is highly dependent on the processing conditions of the fibers. Figure 2h presents the tensile properties of fibers produced at different processing temperatures (*T*_process_) during twin-screw extrusion. It should be noted that elongation is defined as the strain at maximum stress due to the distinctive tensile behavior of PTFE. As *T*_process_ increases from 180 to 220 °C, the tensile strength and modulus decrease from 34.6 and 252 MPa to 13.3 and 42.8 MPa, respectively, while the tensile elongation increases from 20.2 to 38.1%. This is attributed to the lower melt viscosity of PLA at higher *T*_process_, resulting in a lower degree of fibrillation and weaker entanglement of PTFE fibrils. These fibrils are more prone to further fibrillation and slippage under external forces, leading to lower strength and higher elongation. To further investigate, we compared the tensile properties of fibers produced at the same *T*_process_ but with different PTFE-to-PLA ratios, as shown in Fig. S6. The fibers exhibit comparable strength, modulus, and elongation due to similar melt viscosities during twin-screw compounding (Fig. S7), which is practicable in complicate applications.

### Spectral Engineering for Optimal Radiative PTM Properties

**Fig. 3:**
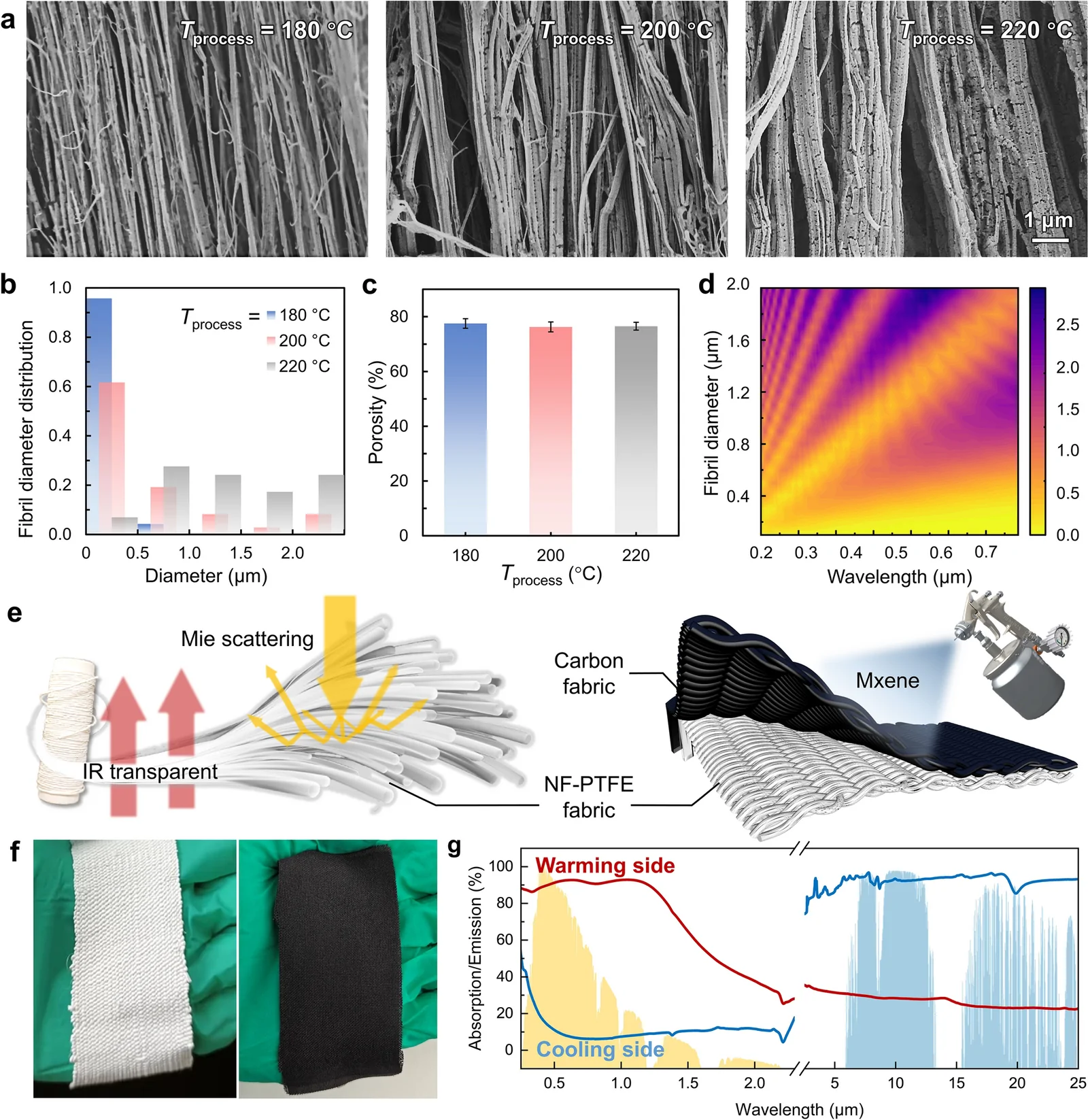
Spectral design and optimization for radiative PTM. **a** SEM images of microporous PTFE fibers processed at different *T*_process_. **b** Porosity of microporous PTFE fibers at different *T*_process_. **c** Diameter distribution of fibrils in PTFE fibers at different *T*_process_. **d** Theoretical scattering efficiency of PTFE fibrils with different diameters in the UV–vis–NIR wavelength range. **e** Schematic of the radiative cooling mechanism of microporous PTFE fiber (left) and fabrication of Janus textile by coupling MXene-coated carbon fabric. **f** Photograph showing the appearance of the prepared Janus textile. **g** Spectral characteristics of the Janus textile, highlighting contrasting absorption/emission on each side

To achieve optimal personal thermal management (PTM) efficiency, it is crucial to manipulate the optical properties of the PTFE textile to ensure strong Mie scattering intensity, which is highly dependent on the fiber microstructure. Fibers with different microfibril diameters can be readily produced by adjusting the degree of fibrillation through variation of the processing temperature (*T*_process_). As shown in Fig. 3a, fibers processed at 180 °C primarily consist of dense nanofibrils, whereas higher *T*_process_ values result in the formation of coarser and sparser microfibrils. Figure 3b demonstrates the fibril diameter distribution: fibrils processed at 180 °C have fine diameters concentrated within 0.5 μm, while increased *T*_process_ leads to a broader range of fibril diameters. At a *T*_process_ of 220 °C, the fibril diameter is evenly distributed across the range of 0.5–2.5 μm. Despite the varied distribution of fibrils sizes, the high porosity (> 75%) remains nearly constant (Fig. 3c). According to theoretical calculations shown in Fig. 3d, the presence of multi-dimensional fibrils at both nano- and micro- scales enables high scattering efficiency across the visible light (VL) spectrum, whereas a narrow sub-micron diameter distribution results in poor scattering intensity. Therefore, microporous PTFE fibers processed at 220 °C were selected for the subsequent fabrication of PTM textiles. Additionally, the fiber exhibit excellent infrared emittance near the atmospheric window and high transparency in the other wavelength range (Fig. S8). Although the optical simulations were conducted using single-fiber models for simplicity, the actual PTFE fibers were woven into a fabric with a plain weaving structure, which can mitigate the anisotropic scattering of individual fibers and yield a more uniform spectral response [54].

We prepared X-Janus textile by laminating the woven PTFE textile with MXene-coated carbon fabric (Fig. S9) by sewing. As a result, a spectral Janus textile, as schematically presented in Fig. 3e, was successfully fabricated (Fig. 3f), displaying the required distinct optical properties on each side. As shown in Fig. 3g, the optimized cooling side of the fabric exhibits an extremely white color with low absorption of 9.6% in the VL and near-IR ranges, reflecting most solar light, while demonstrating exceptionally high emittance of 92.7% in the atmosphere window range. In contrast, the warming side of the fabric shows dark black color with the absorption of 86.7% in the VL range and low IR emittance of 27.2% across the 7–25 μm spectrum. These spectral curves closely match the ideal conditions proposed in Fig. 1c.

### Evaluation of Switchable Passive PTM Performance

**Fig. 4:**
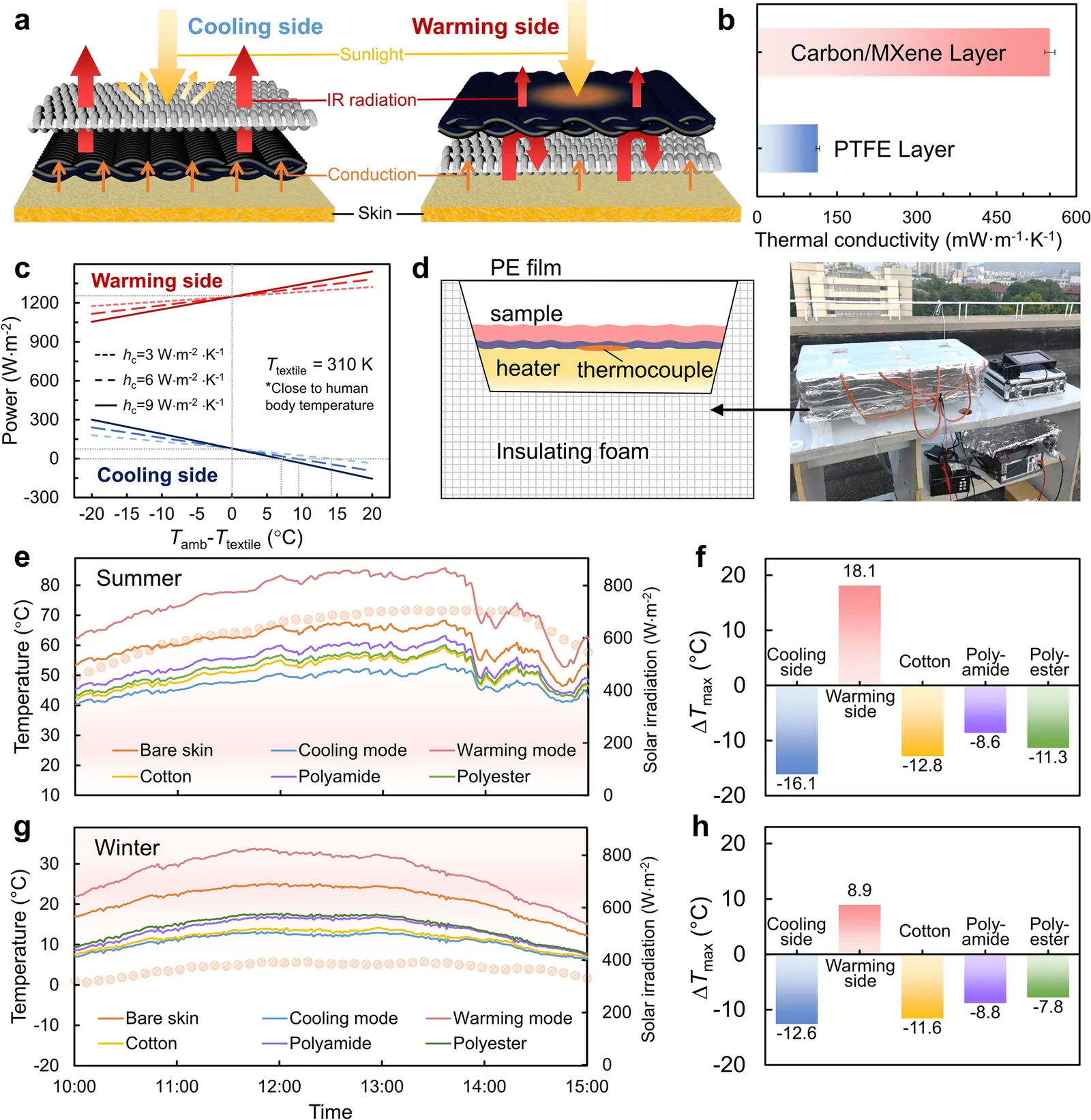
Passive PTM performance of X-Janus textile. **a** Schematic of the PTM mechanism on each side of the X-Janus textile.** b** Thermal conductivity of PTFE fabric and MXene-coated carbon fabric. **c** Calculated cooling/heating power of the Janus textiles as a function of ambient temperature (*T*_amb_) with *T*_textile_ at 36.5 °C. **d** Schematic (left) and photograph (right) of the thermal measurement equipment used to characterize PTM performance. **e** Continuous temperature monitoring and **f** maximum temperature difference of cooling performance between X-Janus textile and commercial textiles under hot summer weather, the inset pink domains suggest the optimal temperature range for human tolerance. **g** Continuous temperature monitoring and **h** maximum temperature difference of PTM performance between X-Janus textile and commercial textiles under cold winter conditions

The detailed PTM mechanism of the X-Janus textile is illustrated in Fig. 4a. For cooling purposes, the PTFE layer is positioned outward to reflect sunlight via strong Mie scattering, while the inner carbon layer acts as a dissipater, transferring heat from the skin to the external environment and outer space through mid-IR emission that penetrates the PTFE layer and the MXene layer can effectively reflect the IR from surroundings. For warming, the carbon/MXene layer is oriented outward to actively absorb solar energy, while the MXene coating reflects thermal radiation from the skin, passively preserving body heat. In addition to thermal radiation, thermal conduction also plays a key role in efficient PTM. As shown in Fig. 4b, the PTFE layer exhibits a low thermal conductivity of 114 mW m^−1^ K^−1^, which insulates against external heat in hot environments and helps retain body warmth in cold conditions (Fig. S10). Conversely, the carbon/MXene layer presents a high thermal conductivity of 550 mW m^−1^ K^−1^, facilitating body heat transfer in hot environments and the conduction of photothermal energy in cold environments. Meanwhile, the air layer between can act as buffer layer that prevent external heat transfer in hot environment and heat loss of human body in cold environment. Therefore, the thermal conduction difference plays an important role in facilitating the heat transfer between textile and human skin, while the thermal radiation is critical in regulation the heat transfer with environments. Theoretical calculations of radiative cooling and heating power, considering different coefficients of non-radiative heat transfer (*h*_c_), are shown in Fig. 4c. The results demonstrate that the cooling side achieves a net cooling power of 77.61 W m^−2^ at thermal equilibrium, and the cooling function is effective when the ambient temperature (*T*_ambient_) exceeds the textile temperature (*T*_textile_), which is close to skin temperature (310 K). Meanwhile, due to the superior photothermal conversion effect of the warming side, its theoretical net heating power reaches 1247 W m^−2^, about 91.6% of the solar constant (1361 W m^−2^).

We evaluated the PTM performance during both hot summer and cold winter days in Jinan, China (36.7°N, 117.0°E), using a bespoke measurement apparatus as shown in Fig. 4d. For comparison, three common textiles including cotton, polyamide, and polyester were also tested. Figure 4e shows the passive cooling performance during the hottest period from 10:00 to 15:00 on sunny summer days. The cooling side of the X-Janus textile resulted in the lowest simulated skin surface temperature among all samples, with a maximum temperature difference (|*ΔT*_max_ |) between the textile and bare skin reaching 16.1 °C. This is 25.8%, 87.5%, and 42.1% higher than cotton, polyamide, and polyester, respectively (Fig. 4f). Figure 4g presents the photothermal heating performance under cold, sunny winter conditions. The warming side of the textile effectively elevated the simulated skin temperature, with a |*ΔT*_max_| of 13.9 °C, while the cooling side and commercial textiles failed to retain warmth and instead decreased skin temperatures. Therefore, by employing the as-prepared X-Janus textile, the simulated skin temperature is maintained below 45 °C on hot summer days and above 15 °C on cold winter days, which is within the human tolerable range and may significantly reduce the risk of health issues caused by extreme weather.

### Self-powered Perception by Triboelectric Effect

**Fig. 5:**
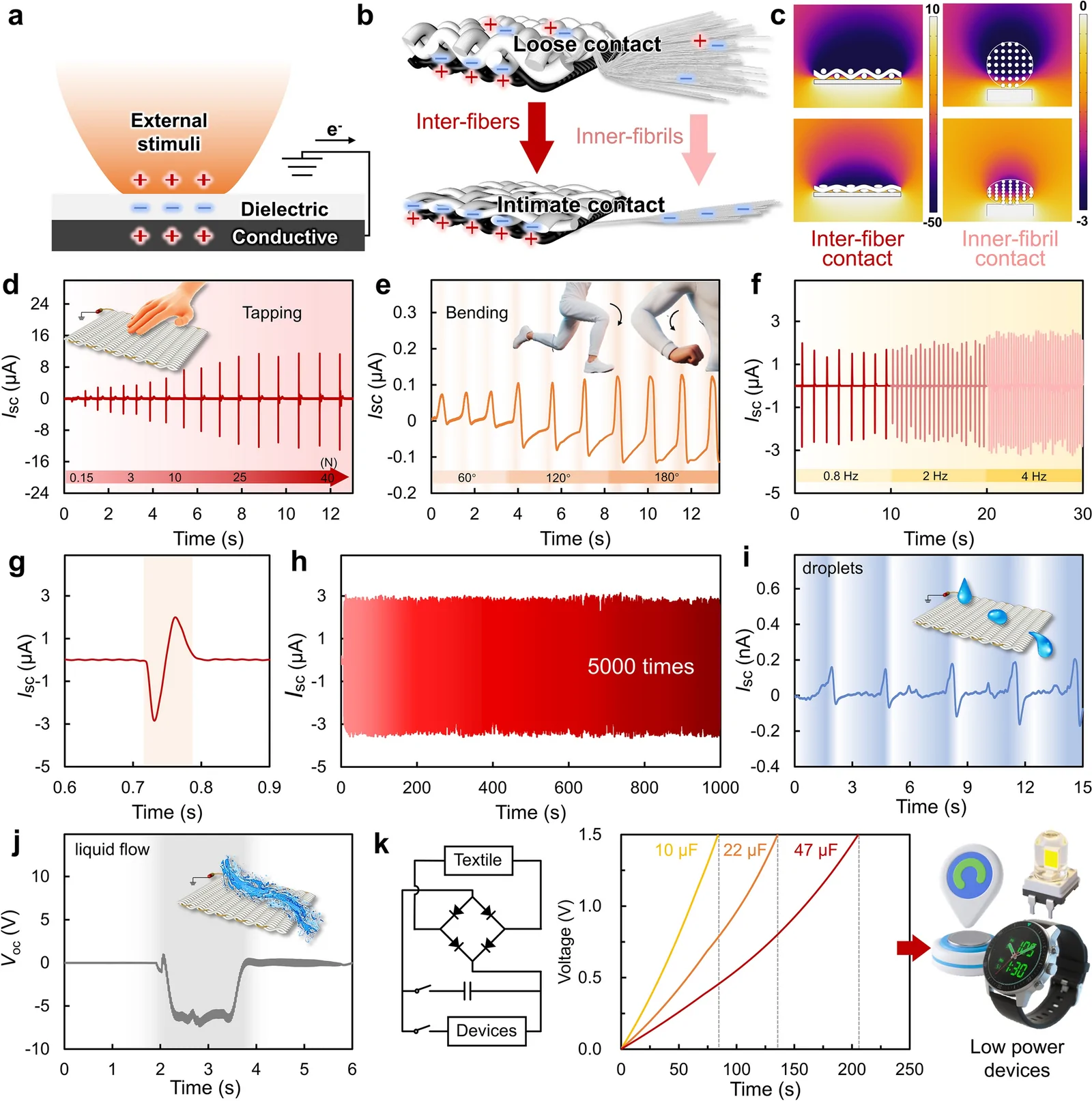
Self-powered perception performance. **a** Schematic of the triboelectric effect-enabled sensing mechanism of the X-Janus textile. **b** Schematic of the multidimensional fiber woven structures enhancing contact electrification. **c** Finite element simulation results showing potential distribution induced by inter-fiber (left) and inner-fibril (right) contact, unit: V. **d** Output current (*I*_sc_) signals generated by hand tapping as a function of different forces. **e** *I*_sc_ signals generated by textile bending as a function of different bending angles. **f** Variation of *I*_sc_ signals with different frequencies (0.8–4 Hz) during contact with cotton fabric. **g** Profile of a single *I*_sc_ signal cycle showing prompt response within 0.1 s. **h** Long-term durability test of stable *I*_sc_ signals over more than five thousand contact-separation cycles. **i** *I*_sc_ signals generated by water droplets of different volumes.** j** *V*_oc_ signals generated by water flow impact. **k** Energy harvesting circuit diagram (left) and voltage–time curves for charging different capacitors via contact with cotton, demonstrating feasibility for powering low-power device

The electrical Janus configuration, comprising a dielectric PTFE layer and a conductive carbon/MXene layer, is ideally suited for single-electrode triboelectric sensors that generate electrical signals in response to external stimuli, as shown in Figs. 5a and S11. As illustrated in Fig. 5b, both macroscopic fiber contacts (inter-fiber) and numerous nanofibril contacts (inner-fibril) become more intimate under pressure, resulting in distinctive contact polarization behavior. Figure 5c presents simulated electric potential distributions for the woven PTFE layer and a single microporous PTFE fiber attached to a conductive layer under different degrees of compressive force, as determined by finite element simulation using a simplified cross-sectional 2D model. The results reveal that different pressing degrees generate varying electric potentials in the upper PTFE layer, which in turn produce characteristic current flows in the grounded conductive layer, serving as signals for different external stimuli. The woven structure of the textile, formed from microporous PTFE fibers composed of fibrillated nanofibrils, enables a hierarchical contact mechanism under external forces, thereby enhancing the textile’s perception capabilities. Therefore, the designed textile can function as a smart sensor to detect deformation or stimuli based on contact-induced triboelectric signals, without the need for additional energy sources.

The triboelectric output performance of the X-Janus textile was evaluated by measuring the generated current under different deformation modes. Figure 5d displays the output current of the textile, placed flat, in response to increasing tapping forces applied by hand. The textile exhibits a sensitive response to very slight contact, with a detectable output at forces as low as 0.15 N, and the output current increases with higher force, reaching a plateau when the force exceeds 25 N. This initial increase is attributed to the growing practical contact area, while the plateau corresponds to the fully compressed state of the porous fiber and woven textile. Furthermore, the textile generates electrical output under varying degrees of bending. As shown in Fig. 5e, increasing the bending angle results in higher output current, reflecting the sensitivity enabled by the hierarchical fibrous structure. Figure 5f shows the output current as a function of tapping frequency to show the adaptivity, the textile provides stable responses within the frequency range of 0.8–4 Hz that aligns well with typical human movement frequencies. Figure 5g demonstrates the textile’s prompt response to external stimuli, with a reaction time within 0.1 s. These results indicate that the textile can respond quickly to various deformation modes like pressing and bending during different activities. Figure 5h shows the electrical response after more than 5,000 tapping cycles, further demonstrating the outstanding stability and durability of the triboelectric effect in the X-Janus textile.

Notably, beyond deformation detection, the triboelectric effect also endows the textile with excellent environmental perception capabilities. For example, as shown in Fig. 5i, when water droplets drip and slide off the PTFE layer, specific electric current signals are detected in the conductive layer, corresponding to variations in droplet volume and frequency. Moreover, the textile can detect not only individual droplets but also continuous water flow impacting the PTFE surface (Fig. 5j). This water contact sensitivity arises from triboelectric charge induction between the droplet and polymer surface, generating electric current signals that reflect information such as droplet volume and dripping frequency during rainy weather, thereby providing early warning of sudden weather changes for human safety. In addition to the aforementioned sensing properties, the electricity generated by the significant triboelectric effect during motion-contact can be harvested to supply low-power devices. As illustrated in the circuit diagram in Fig. 5k, the alternating current generated by the X-Janus textile is rectified to direct current to charge a capacitor. Using cotton fabric as the contact material and a contact frequency of 4 Hz (Fig. S12), capacitors of 10, 22, and 47 μF can be charged to 1.5 V within 83.5, 135, and 205 s, respectively, meeting the energy requirements of various low-power devices such as positioning devices, LEDs, and electronic timers. Therefore, the X-Janus textile achieves self-powered sensing via the triboelectric effect (Video S1), enabling real-time detection of human motion and water droplet impacts while harvesting energy to power devices, demonstrating its potential for smart wearable systems, environmental monitoring, and sustainable energy solutions.

### Wearing Comfort and Comprehensive Protection Properties

**Fig. 6:**
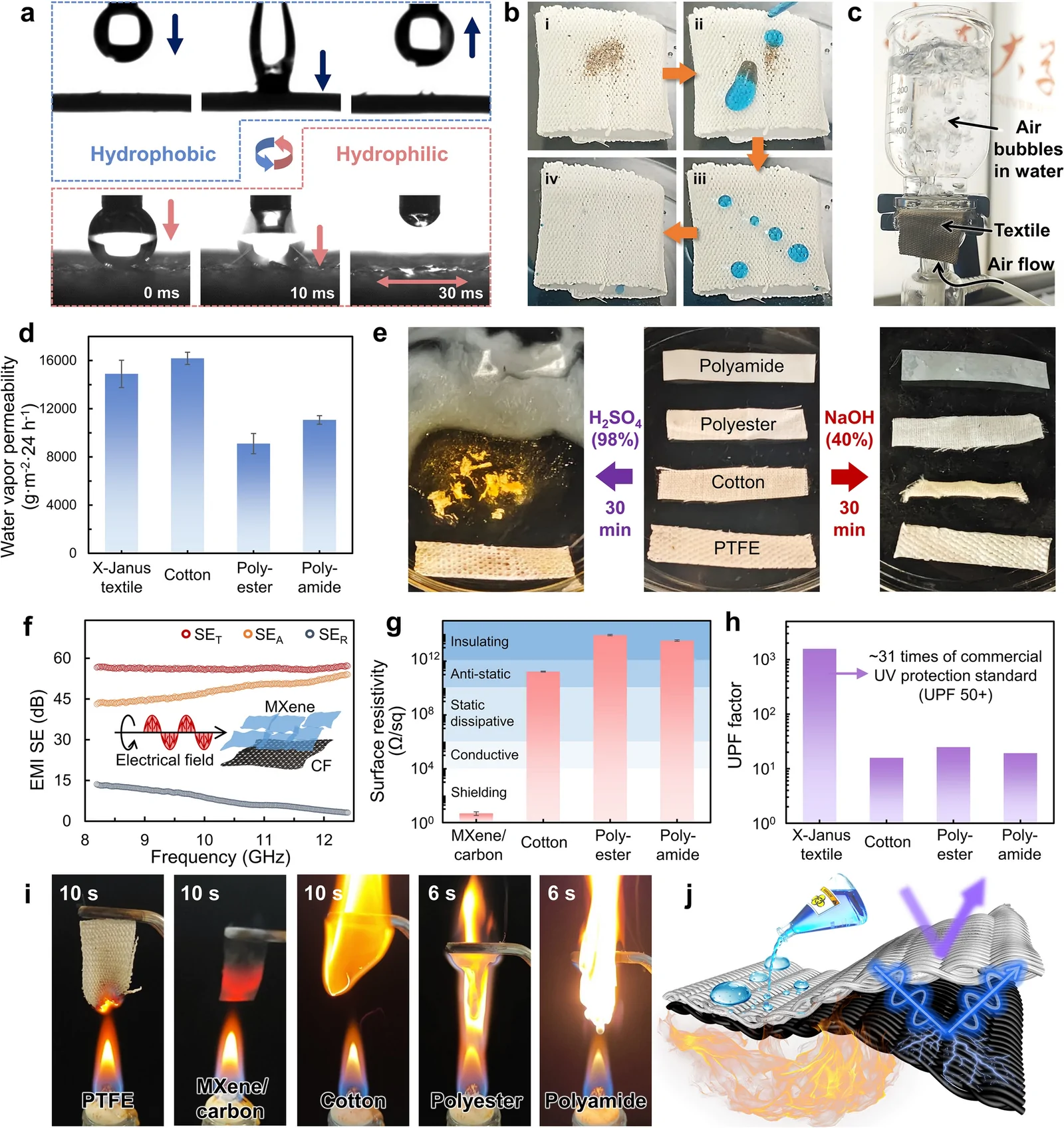
Wearing comfort and multifunctional protection properties. **a** Water contact measurement by placing a 5 μL droplet on the X-Janus textile, demonstrating opposite wetting behaviors on each side. **b** Illustration of self-cleaning ability by dripping water onto a contaminated PTFE surface. **c** Demonstration of waterproofness and breathability of the X-Janus textile. **d** Water vapor permeability test showing advanced perspiration transmission compared to other commercial textiles. **e** Chemical resistance test by immersing textiles in strong acid and alkali solutions for 30 min. **f** EMI shielding effectiveness of the X-Janus textile, indicating over 99.9997% of EMI can be blocked. **g** Anti-static performance reflected by the ultralow surface resistivity of the MXene/carbon fabric. **h** UPF factor comparison between textiles, demonstrating superior UV shielding performance of the X-Janus textile. **i** Flame retardancy test by vertically placing textiles over a flame for 10 s. **j** Schematic of the multifunctional protection properties of the X-Janus textile

The designed textile also exhibits a remarkable Janus feature in wettability. As demonstrated in Fig. 6a, a 5 μL water droplet cannot wet the single PTFE fiber even after firm contact, due to the outstanding hydrophobicity of PTFE. In contrast, the opposite side of MXene-coated carbon textile shows completely different wetting behavior, with the droplet instantly spreading into the textile within 30 ms, demonstrating superior hydrophilicity. This Janus design, with reverse wettability on each side, further enhances thermal and moisture comfort for the wearer. For example, when the cooling side is used in hot environments, the carbon/MXene layer in close contact with the skin can efficiently absorb sweat, promoting evaporation and significantly contributing to body cooling via endothermic phase change. Conversely, when the warming side is used in cold environments, the PTFE layer is placed inside, in contact with the skin, to prevent external cold moisture from affecting the body, thereby maintaining dryness and warmth. Moreover, the hydrophobic PTFE layer provides excellent waterproofness during heavy rain and self-cleaning functionality, which is particularly useful for outdoor applications (Fig. 6b). As visualized in Fig. 6c, the textile demonstrates outstanding waterproofness by withstanding water from above, while continuous air transmission through the textile generates numerous bubbles, reflecting its remarkable breathability. The textile has a water vapor transmittance of 14,897 g m^−2^ (24 h)^−1^, comparable to commercial cotton textiles and significantly higher than polyester and polyamide textiles (Fig. 6d). This breathability enables necessary heat exchange for favorable wearing performance, ensuring optimal human comfort.

In practical scenarios, the X-Janus textile also provides comprehensive protection properties for diverse working conditions. First, the firmly woven textile offers extraordinary chemical protection due to the well-known chemical inertness of PTFE. In Fig. 6e, we demonstrated the chemical resistance of our textile and other commercial textiles by immersing them in harsh acid and alkaline solutions for 30 min. Remarkably, the commercial textiles were quickly decomposed by the acid solution, and cotton was destroyed by the alkali. In stark contrast, the PTFE layer remained intact except for slight yellowing in acid, likely due to the deterioration of residual PLA in the PTFE fibers. Additionally, as shown in Fig. 6f, the conductive carbon/MXene layer provides outstanding EMI shielding effectiveness (SE) as high as 56 dB across the entire X-band (8.2–12.4 GHz), indicating that more than 99.9997% of hazardous EMI waves can be blocked from reaching the human body. Regarding the EMI shielding mechanism, absorption (SE_A_) is significantly higher than reflection (SE_R_) due to the multi-reflection (Fig. S13), especially in the high-frequency region, which greatly diminishes secondary radiation. The high conductivity also enables a surface resistance of the MXene-coated carbon layer is measured at 4.8 Ω sq^−1^, classifying it as a conductive textile with excellent anti-static performance (Fig. 6g). This combination offsets the intrinsic drawback of PTFE, which is prone to generating static hazards. Furthermore, in Figs. 6h and S14, the X-Janus textile exhibits excellent ultraviolet (UV) protection due to its low UV transmittance. The ultraviolet protection factor (UPF) of the X-Janus textile is calculated to be above 1,500, far exceeding commercial cotton, polyester, and polyamide textiles, and approximately 31 times higher than the highest standard (UPF 50 +) for commercial UV-protective textiles. Lastly, the flame retardancy of the textiles was assessed by vertical burning tests. As shown in Fig. 6i and Video S2, when the textile is held above an open flame for 10 s, only minimal burning with faint smoldering occurs, and it extinguishes instantly once removed from the flame, clearly demonstrating outstanding flame resistance. The PTFE layer resists flame due to stable carbon–fluorine bonds, while the carbon/MXene layer relies on the formation of a synergistic ceramic-like char layer to suppress combustion by isolating oxygen and dissipating heat. In stark contrast, cotton textiles blacken and char when held above a flame for 10 s, continuing to burn with self-sustaining combustion even after the flame is removed. Meanwhile, polyester and polyamide textiles ignite violently and melt into dripping streams upon exposure to flame, being entirely consumed within 6 s as molten droplets fuel rapid combustion. In short, this comprehensive protective textile integrates breathability, hydrophobicity, chemical resistance, EMI and UV shielding, anti-static properties, and flame retardancy with rapid self-extinguishing, enabling synergistic multifunctionality critical for extreme environments. It offers a versatile solution for hazardous industrial operations, aerospace, and electronic protection, establishing a new benchmark for multifunctional textiles that combine comprehensive safety with advanced functional performance.

## Conclusions

The X-Janus textile represents a paradigm shift in multifunctional textiles by integrating passive thermal management, self-powered sensing, and comprehensive protection through a multifaceted Janus design. Central to its performance is the shear-induced fibrillation methodology, an innovative technique that enables the continuous production of microporous PTFE fibers with multidimensional nano- to microscale fibrils. These fibrils form a unidirectional, fibrous structure that enhances Mie scattering for radiative cooling. The woven PTFE side reflects over 90% of solar radiation while maintaining 85% mid-IR emissivity, facilitating efficient heat dissipation to the environment (resulting in a 16.1 °C temperature drop in summer). Conversely, the MXene-coated carbon fabric side achieves 92% visible light absorption for photothermal conversion and 90% IR reflection, effectively retaining body heat and producing a 13.9 °C temperature increase in cold conditions. The electrical Janus interface, formed by the dielectric PTFE and conductive MXene/carbon layers, exploits triboelectric effects to generate electrical signals. The hierarchical fibrous structure increases the contact area during deformation, enabling sensitive detection of mechanical stimuli (e.g., tapping, bending) within 0.1 s and stable performance over 5,000 cycles. Additionally, the triboelectric effect allows for energy harvesting, enabling capacitors to be charged and low-voltage devices to be powered without external batteries. The wetting Janus design leverages the hydrophobicity of PTFE and the hydrophilicity of the MXene/carbon fabric to achieve waterproof breathability, promoting sweat evaporation while blocking external moisture. Mechanistically, the chemical inertness of the PTFE layer and the high conductivity of the carbon/MXene layer provide comprehensive protection, including 56 dB EMI shielding in the X-band, a UPF greater than 1,500 for UV resistance, and outstanding flame retardancy. This work demonstrates that a multifaceted Janus design can integrate emerging technologies to synergistically address critical challenges in comfort, safety, adaptability, and sustainability, establishing a new benchmark for advanced multifunctional textiles.

## Supplementary Information

Below is the link to the electronic supplementary material.

Supplementary file1 (DOCX 5569 KB)

Supplementary file2 (MP4 3418 KB)

Supplementary file3 (MP4 1670 KB)

## References

[1] K. Chen, E. de Schrijver, S. Sivaraj, F. Sera, N. Scovronick et al., Impact of population aging on future temperature-related mortality at different global warming levels. Nat. Commun. **15**(1), 1796 (2024). 10.1038/s41467-024-45901-z 38413648 10.1038/s41467-024-45901-z PMC10899213

[2] Y. Wu, S. Li, Q. Zhao, B. Wen, A. Gasparrini et al., Global, regional, and national burden of mortality associated with short-term temperature variability from 2000–19: a three-stage modelling study. Lancet Planet. Health **6**(5), e410–e421 (2022). 10.1016/S2542-5196(22)00073-0 35550080 10.1016/S2542-5196(22)00073-0 PMC9177161

[3] J. Sun, Energy supply and influencing factors of mountain marathon runners from Baiyin marathon accident in China. Sci. Rep. **12**(1), 8179 (2022). 10.1038/s41598-022-12403-1 35581400 10.1038/s41598-022-12403-1 PMC9112246

[4] Y. Peng, Y. Cui, Advanced textiles for personal thermal management and energy. Joule **4**(4), 724–742 (2020). 10.1016/j.joule.2020.02.011

[5] S. Hong, Y. Gu, J.K. Seo, J. Wang, P. Liu et al., Wearable thermoelectrics for personalized thermoregulation. Sci. Adv. **5**(5), eaaw0536 (2019). 10.1126/sciadv.aaw0536 31114803 10.1126/sciadv.aaw0536 PMC6524982

[6] J. Chai, G. Wang, G. Wang, R. Shao, J. Zhao et al., Porous and conductive fiber woven textile for multi-functional protection, personal warmth, and intelligent motion/temperature perception. Adv. Funct. Mater. **35**(10), 2416428 (2025). 10.1002/adfm.202416428

[7] L. Lou, Y. Zhou, Y. Yan, Y. Hong, J. Fan, Wearable cooling and dehumidifying system for personal protective equipment (PPE). Energy Build. **276**, 112510 (2022). 10.1016/j.enbuild.2022.112510

[8] G. Chen, X. Xiao, X. Zhao, T. Tat, M. Bick et al., Electronic textiles for wearable point-of-care systems. Chem. Rev. **122**(3), 3259–3291 (2022). 10.1021/acs.chemrev.1c00502 34939791 10.1021/acs.chemrev.1c00502

[9] S. Márquez-Sánchez, I. Campero-Jurado, J. Herrera-Santos, S. Rodríguez, J.M. Corchado, Intelligent platform based on smart PPE for safety in workplaces. Sensors **21**(14), 4652 (2021). 10.3390/s21144652 34300392 10.3390/s21144652 PMC8309589

[10] Y. Shi, F. Wang, J. Tian, S. Li, E. Fu et al., Self-powered electro-tactile system for virtual tactile experiences. Sci. Adv. **7**(6), eabe2943 (2021). 10.1126/sciadv.abe2943 33536215 10.1126/sciadv.abe2943 PMC7857682

[11] D. Yang, H.K. Nam, Y. Lee, S. Kwon, J. Lee et al., Laser-induced graphene smart textiles for future space suits and telescopes. Adv. Funct. Mater. **35**(1), 2411257 (2025). 10.1002/adfm.202411257

[12] L. Lou, K. Chen, J. Fan, Advanced materials for personal thermal and moisture management of health care workers wearing PPE. Mater. Sci. Eng. R. Rep. **146**, 100639 (2021). 10.1016/j.mser.2021.100639 34803231 10.1016/j.mser.2021.100639 PMC8590464

[13] Y. Wei, L. Zhang, F. Bernasconi, T. Wu, Y. Li et al., Temperature-responsive resonator metafabrics for self-adaptive thermoregulation. Adv. Funct. Mater. **35**(40), 2422485 (2025). 10.1002/adfm.202422485

[14] C. Guo, C. Li, Z. Qiao, C. Lei, Z. Ju et al., Crack-resistant and self-healable passive radiative cooling silicone compounds. Adv. Mater. **37**(14), 2500738 (2025). 10.1002/adma.202500738 10.1002/adma.202500738 40018766

[15] Q. Ye, Y. Huang, B. Yao, Z. Chen, C. Shi et al., Radiative coupled evaporation cooling hydrogel for above-ambient heat dissipation and flame retardancy. Nano-Micro Lett. **18**(1), 50 (2025). 10.1007/s40820-025-01903-0 10.1007/s40820-025-01903-0 PMC12401836 40889007

[16] N. Guo, L. Yu, C. Shi, H. Yan, M. Chen, A facile and effective design for dynamic thermal management based on synchronous solar and thermal radiation regulation. Nano Lett. **24**(4), 1447–1453 (2024). 10.1021/acs.nanolett.3c04996 38252892 10.1021/acs.nanolett.3c04996

[17] J. Sui, S. Jiang, J. Peng, Z. Kang, J. Fan, Cellular core/sheath filaments with thermoresponsive vacuum cavities for prolonged passive temperature-adaptive thermoregulation. Adv. Sci. **12**(8), 2412448 (2025). 10.1002/advs.202412448 10.1002/advs.202412448 PMC11848530 39764738

[18] Q. Zhang, H. Cheng, S. Zhang, Y. Li, Z. Li et al., Advancements and challenges in thermoregulating textiles: smart clothing for enhanced personal thermal management. Chem. Eng. J. **488**, 151040 (2024). 10.1016/j.cej.2024.151040

[19] H. Liu, F. Zhou, X. Shi, K. Sun, Y. Kou et al., A thermoregulatory flexible phase change nonwoven for all-season high-efficiency wearable thermal management. Nano-Micro Lett. **15**(1), 29 (2023). 10.1007/s40820-022-00991-6 10.1007/s40820-022-00991-6 PMC9813330 36598606

[20] B. Gu, Z. Dai, H. Pan, D. Zhao, Integration of prolonged phase-change thermal storage material and radiative cooling textile for personal thermal management. Chem. Eng. J. **493**, 152637 (2024). 10.1016/j.cej.2024.152637

[21] D. Li, W. Liu, T. Peng, Y. Liu, L. Zhong et al., Janus textile: advancing wearable technology for autonomous sweat management and beyond. Small **21**(13), 2409730 (2025). 10.1002/smll.202409730 10.1002/smll.202409730 40042440

[22] Y.-Y. Xiao, Z.-C. Jiang, X. Tong, Y. Zhao, Biomimetic locomotion of electrically powered “Janus” soft robots using a liquid crystal polymer. Adv. Mater. **31**(36), 1903452 (2019). 10.1002/adma.201903452 10.1002/adma.201903452 31298439

[23] Z. Wu, K. Yin, J. Wu, Z. Zhu, J.-A. Duan et al., Recent advances in femtosecond laser-structured Janus membranes with asymmetric surface wettability. Nanoscale **13**(4), 2209–2226 (2021). 10.1039/D0NR06639G 33480955 10.1039/d0nr06639g

[24] Y. Zhang, J. Fu, Y. Ding, A.A. Babar, X. Song et al., Thermal and moisture managing E-textiles enabled by Janus hierarchical gradient honeycombs. Adv. Mater. **36**(13), 2311633 (2024). 10.1002/adma.202311633 10.1002/adma.202311633 38112378

[25] C. Jeong, K.Y. Kwon, D. Wu, Y. Fu, Y.-S. Ye et al., Reconfigurable double-sided smart textile circuit with liquid metal. Mater. Horiz. **12**(15), 5710–5722 (2025). 10.1039/D5MH00462D 40370052 10.1039/d5mh00462d

[26] P.-C. Hsu, C. Liu, A.Y. Song, Z. Zhang, Y. Peng et al., A dual-mode textile for human body radiative heating and cooling. Sci. Adv. **3**(11), e1700895 (2017). 10.1126/sciadv.1700895 29296678 10.1126/sciadv.1700895 PMC5688747

[27] K. Zhu, H. Yao, J. Song, Q. Liao, S. He et al., Temperature-adaptive dual-modal photonic textiles for thermal management. Sci. Adv. **10**(41), eadr2062 (2024). 10.1126/sciadv.adr2062 39383222 10.1126/sciadv.adr2062 PMC11463281

[28] K. Li, M. Li, C. Lin, G. Liu, Y. Li et al., A janus textile capable of radiative subambient cooling and warming for multi-scenario personal thermal management. Small **19**(19), 2206149 (2023). 10.1002/smll.202206149 10.1002/smll.202206149 36807770

[29] L. Yuan, S. Jia, S. Shao, H.M. Asfahan, X. Li, A self-cleaning janus textile for highly efficient heating and cooling management. Nano Lett. **25**(19), 8019–8026 (2025). 10.1021/acs.nanolett.5c01738 40314160 10.1021/acs.nanolett.5c01738

[30] M. Feng, S. Feng, T. Yu, S. Zhu, H. Cai et al., Versatile and comfortable janus fabrics for switchable personal thermal management and electromagnetic interference shielding. Adv. Fiber Mater. **6**(3), 911–924 (2024). 10.1007/s42765-024-00393-w

[31] C. Chen, B. Zhao, R. Wang, Z. He, J.-L. Wang et al., Janus helical ribbon structure of ordered nanowire films for flexible solar thermoelectric devices. Adv. Mater. **34**(44), 2206364 (2022). 10.1002/adma.202206364 10.1002/adma.202206364 36120802

[32] C. Zhi, S. Shi, S. Zhang, Y. Si, J. Yang et al., Bioinspired all-fibrous directional moisture-wicking electronic skins for biomechanical energy harvesting and all-range health sensing. Nano-Micro Lett. **15**(1), 60 (2023). 10.1007/s40820-023-01028-2 10.1007/s40820-023-01028-2 PMC9981859 36864316

[33] R. Shao, G. Wang, J. Chai, J. Lin, G. Zhao et al., Multifunctional janus-structured polytetrafluoroethylene-carbon nanotube-Fe_3_O_4_/MXene membranes for enhanced EMI shielding and thermal management. Nano-Micro Lett. **17**(1), 136 (2025). 10.1007/s40820-025-01647-x 10.1007/s40820-025-01647-x PMC11802968 39912994

[34] Y. Yao, Y. Guo, X. Li, J. Yu, B. Ding, Asymmetric wettable, waterproof, and breathable nanofibrous membranes for wound dressings. ACS Appl. Bio Mater. **4**(4), 3287–3293 (2021). 10.1021/acsabm.0c01624 10.1021/acsabm.0c01624 35014415

[35] Y. Ni, B. Li, C. Chu, S. Wang, Y. Jia et al., One-step fabrication of ultrathin porous Janus membrane within seconds for waterproof and breathable electronic skin. Sci. Bull. **70**(5), 712–721 (2025). 10.1016/j.scib.2024.12.040 10.1016/j.scib.2024.12.040 39837718

[36] L. Lao, D. Shou, Y.S. Wu, J.T. Fan, Skin-like” fabric for personal moisture management. Sci. Adv. **6**(14), eaaz0013 (2020). 10.1126/sciadv.aaz0013 32284976 10.1126/sciadv.aaz0013 PMC7124935

[37] C. Fan, Y. Zhang, Z. Long, A. Mensah, Q. Wang et al., Dynamically tunable subambient daytime radiative cooling metafabric with Janus wettability. Adv. Funct. Mater. **33**(29), 2300794 (2023). 10.1002/adfm.202300794

[38] T. Yang, S. Wang, H. Yang, H. Gui, Y. Du et al., Temperature-triggered dynamic Janus fabrics for smart directional water transport. Adv. Funct. Mater. **33**(18), 2214183 (2023). 10.1002/adfm.202214183

[39] S. Zeng, S. Pian, M. Su, Z. Wang, M. Wu et al., Hierarchical-morphology metafabric for scalable passive daytime radiative cooling. Science **373**(6555), 692–696 (2021). 10.1126/science.abi5484 34353954 10.1126/science.abi5484

[40] J. Li, Y. Fu, J. Zhou, K. Yao, X. Ma et al., Ultrathin, soft, radiative cooling interfaces for advanced thermal management in skin electronics. Sci. Adv. **9**(14), eadg1837 (2023). 10.1126/sciadv.adg1837 37027471 10.1126/sciadv.adg1837 PMC10081843

[41] J. Wu, J. He, K. Yin, Z. Zhu, S. Xiao et al., Robust hierarchical porous PTFE film fabricated *via* femtosecond laser for self-cleaning passive cooling. Nano Lett. **21**(10), 4209–4216 (2021). 10.1021/acs.nanolett.1c00038 33970640 10.1021/acs.nanolett.1c00038

[42] Y. Zhang, X. Du, J. Huangfu, K. Chen, X. Han et al., Self-cleaning PTFE nanofiber membrane for long-term passive daytime radiative cooling. Chem. Eng. J. **490**, 151831 (2024). 10.1016/j.cej.2024.151831

[43] T. Tian, X. Wei, A. Elhassan, J. Yu, Z. Li et al., Highly flexible, efficient, and wearable infrared radiation heating carbon fabric. Chem. Eng. J. **417**, 128114 (2021). 10.1016/j.cej.2020.128114

[44] M. Chen, X. Jiang, J. Huang, J. Yang, J. Wu et al., Flexible wearable Ti_3_C_2_T_*x*_ composite carbon fabric textile with infrared stealth and electromagnetic interference shielding effect. Adv. Opt. Mater. **12**(4), 2301694 (2024). 10.1002/adom.202301694

[45] B. Shao, M.-H. Lu, T.-C. Wu, W.-C. Peng, T.-Y. Ko et al., Large-area, untethered, metamorphic, and omnidirectionally stretchable multiplexing self-powered triboelectric skins. Nat. Commun. **15**(1), 1238 (2024). 10.1038/s41467-024-45611-6 38336848 10.1038/s41467-024-45611-6 PMC10858173

[46] J. Zhao, Y. Peng, P. Hu, X. Hu, X. Su et al., Knot-patterned treble-weaving smart electronic textiles with advanced thermal and moisture regulation for seamless motion monitoring. Adv. Funct. Mater. **35**(35), 2501912 (2025). 10.1002/adfm.202501912

[47] J. Zhang, H. Dong, Y. Yu, S. Yin, M. Cao et al., An eco-friendly and multifunctional textile integrating radiative heating and cooling for large-temperature-variation personal thermal management. Adv. Funct. Mater. **35**(24), 2419777 (2025). 10.1002/adfm.202419777

[48] H. Zhao, Q. Sun, J. Zhou, X. Deng, J. Cui, Switchable cavitation in silicone coatings for energy-saving cooling and heating. Adv. Mater. **32**(29), 2000870 (2020). 10.1002/adma.202000870 10.1002/adma.202000870 32500529

[49] P. Yang, J. He, Y. Ju, Q. Zhang, Y. Wu et al., Dual-mode integrated Janus films with highly efficient NaH_2_PO_2_-enhanced infrared radiative cooling and solar heating for year-round thermal management. Adv. Sci. **10**(7), 2206176 (2023). 10.1002/advs.202206176 10.1002/advs.202206176 PMC9982563 36638249

[50] N. Cheng, Z. Wang, Y. Lin, X. Li, Y. Zhang et al., Breathable dual-mode leather-like nanotextile for efficient daytime radiative cooling and heating. Adv. Mater. **36**(33), 2403223 (2024). 10.1002/adma.202403223 10.1002/adma.202403223 38896500

[51] J. Chai, G. Wang, J. Zhao, G. Wang, C. Wei et al., Robust, breathable, and chemical-resistant polytetrafluoroethylene (PTFE) films achieved by novel *in situ* fibrillation strategy for high-performance triboelectric nanogenerators. Nano Res. **17**(3), 1942–1951 (2024). 10.1007/s12274-023-6147-3

[52] J. Zhao, G. Wang, Z. Chen, Y. Huang, C. Wang et al., Microcellular injection molded outstanding oleophilic and sound-insulating PP/PTFE nanocomposite foam. Compos. Part B Eng. **215**, 108786 (2021). 10.1016/j.compositesb.2021.108786

[53] J. Chai, G. Wang, A. Zhang, G. Zhao, C. B. Park, Superhydrophobic nanofibrous polytetrafluoroethylene and composite membranes with tunable adhesion for micro-droplet manipulation, self-cleaning and oil/water separation. J. Environ. Chem. Eng. **12**(2), 112355 (2024). 10.1016/j.jece.2024.112355

[54] M. Chen, D. Pang, J. Mandal, X. Chen, H. Yan, et al., Designing mesoporous photonic structures for high-performance passive daytime radiative cooling, Nano Lett. **21**, 1412–1418 (2021). 10.1021/acs.nanolett.0c04241. 10.1021/acs.nanolett.0c04241 33524258

