# Supplementary material for: Multifaceted Janus Textile Simultaneously Achieving Self-Sustainable Thermal Management, Perception, and Protection
Source: Nanomicro Lett. 2026 Jan 13;18:205. doi: 10.1007/s40820-025-02030-6 (PMC12799878; doi:10.1007/s40820-025-02030-6)
Supplement: Supplementary file 1 — Supplementary file1 (DOCX 5569 KB) [file 40820_2025_2030_MOESM1_ESM.docx]

Supporting Information for

**Multifaceted Janus Textile Simultaneously Achieving Self-Sustainable Thermal Management, Perception, and Protection**

Jialong Chai^1,2,3^, Guilong Wang^1,2,^*, Runze Shao^1,2^, Lin Ni^1,2^, Guoqun Zhao^1,2^, Jintu Fan^3^

^1^ State key Laboratory of Advanced Equipment and Technology for Metal Forming; Shandong University, Jinan, Shandong 250061, P. R. China

^2^ Key Laboratory for Liquid-Solid Structural Evolution and Processing of Materials (Ministry of Education), Shandong University, Jinan, Shandong 250061, P. R. China

^3^ School of Fashion and Textiles, The Hong Kong Polytechnic University, Hung Hom, Kowloon, Hong Kong 999077, P. R. China

*Corresponding author. E-mail: [guilong@sdu.edu.cn](mailto:guilong@sdu.edu.cn) (Guilong Wang)

**Note S1 Preparation of MXene coated carbon fabric**

The solution of few-layer Ti_3_C_2_T_x_ MXene was prepared using a minimal delamination method. First, 3.2 g of LiF were dissolved in 40 mL of 9 M HCl and stirred for 1 h. Then, Ti_3_AlC_2_ powder was slowly added to the mixture and stirred at 40 °C for 24 h. The resulting dispersion was repeatedly washed with deionized water until the pH of the supernatant was greater than 6. The dispersion was sonicated for 40 minutes under nitrogen atmosphere. Subsequently, the mixture was centrifuged at 3500 rpm for 1 h, and the black supernatant of few-layer MXene dispersion was obtained.

Prior to spray coating, the carbon fabric was cleaned by 5M HCl aqueous solution, deionized water, and ethanol in turn, followed by drying in air at room temperature. Then the MXene solution was repeatedly sprayed on the surface of single side carbon fabric wet film via a spray gun. For each time of coating, evenly spray the solution carbon fabric for 30 seconds, and then carry out the drying process by a hair dryer. Repeat the spraying operation 30 times.

**Note S2 Calculation of net cooling and heating power of X-Janus textile**

There are four basic heat exchanges: radiation, conduction, convection, and sweat evaporation. When the dual-mode nanotextile is exposed to a clear sky, considering all heat exchange processes, the theoretical cooling power ($P_{cooling}$) and heating power ($P_{heating}$) of X-Janus textile can be respectively calculated based on energy balance as follows:

$$P_{cooling}=P_{rad}\left( T \right)-P_{sun}-P_{atm}\left( T_{atm} \right)-P_{non-rad}$$

$$P_{heating}=P_{sun}+P_{atm}\left( T_{atm} \right)-P_{rad}\left( T \right)+P_{non-rad}$$

$P_{rad}\left( T \right)$ is the power radiated out by the X-Janus textile at temperature T, which can be defined as:

$$P_{rad}(T)=2\pi\int_{0}^{\frac{\pi}{2}} d\theta sin\theta cos\theta\int_{2.5\mu m}^{25\mu m} I_{BB}\left( T,\lambda\right)\varepsilon\left( \lambda,\theta\right)d\lambda$$

$$I_{BB}\left( T,\lambda\right)=\frac{2hc^{2}}{\lambda^{5}}\frac{1}{e^{\frac{hc}{\lambda k_{B}T}}-1}$$

where $\varepsilon\left( \lambda,\theta\right)$ is the spectral and angular emissivity of the textile, $h$ is Planck’s constant, $k_{B}$ is Boltzmann constant, and $c$ is the speed of light.

Atmospheric thermal radiation absorbed by the textile can be calculated as follows:

$$P_{atm}(T_{amb})=2\pi\int_{0}^{\frac{\pi}{2}} d\theta sin\theta cos\theta\int_{2.5\mu m}^{25\mu m} I_{BB}\left( T,\lambda\right)\varepsilon\left( \lambda,\theta\right)\varepsilon_{atm}\left( \lambda,\theta\right)d\lambda$$

where $\varepsilon_{atm}\left( \lambda,\theta\right)$ is the spectral and angular emissivity of the atmosphere, which can be defined as $\varepsilon_{atm}\left( \lambda,\theta\right)=1-{t(\lambda)}^{\frac{1}{cos\theta}}$. Here, $t(\lambda)$ is the atmospheric transmittance in the zenith direction, which can be obtained from MODTRAN.

The solar irradiance absorbed by textile ($P_{sun}$) is defined as:

$$P_{rad}(T)=\int_{0.3\mu m}^{2.5\mu m} I_{solar}\left( \lambda\right)\varepsilon_{solar}\left( \lambda\right)d\lambda$$

where $I_{solar}\left( \lambda\right)$ is solar spectral intensity corresponding to wavelength $\lambda$; $\varepsilon_{solar}\left( \lambda\right)$ is the absorption of solar by textile.

the non-radiative power lost ($P_{non-rad}$) due to convection and conduction is defined as:

$$P_{non-rad}=h_{c}(T_{amb}-T)$$

where $h_{c}$ is the non-radiative heat transfer coefficient, which are chosen as 0, 3, 6, 9 W⋅m^-2^⋅K^-1^ in this work.

**Note S3** **Calculation of EMI SE and power parameters**

The EMI SE and power parameters of the samples were calculated based on S-parameters:

$${SE}_{R}=-10\log(1-\left| S_{11}^{2} \right|)$$

$${SE}_{A}=-10\log(\frac{\left| S_{21}^{2} \right|}{1-\left| S_{11}^{2} \right|})$$

$${SE}_{T}={SE}_{R}+{SE}_{A}+{SE}_{M}$$

where $S_{11}$ and $S_{21}$ are scattering parameters. ${SE}_{T}$ represents the total EMI SE, ${SE}_{R}$ denotes the reflection, ${SE}_{A}$ indicates the absorption, and the ${SE}_{M}$ is negligible if the ${SE}_{T}$ is greater than 10 dB.

The parameters $R$, $A$, and $T$ refer to the power coefficients of reflection, absorption, and transmission respectively, can be calculated as follows:

$$R=\left| S_{11}^{2} \right|$$

$$T=\left| S_{21}^{2} \right|$$

$$A=1-R-T$$

The EMI shielding efficiency (%), referring to the capability to block waves in terms of percentage, is obtained using the following equation:

$$Shielding effectiveness \left( \% \right)=100\%-\left( \frac{1}{{10}^{\frac{{SE}_{T}}{10}}} \right)\times100\%$$

**Supplementary Figures**

**
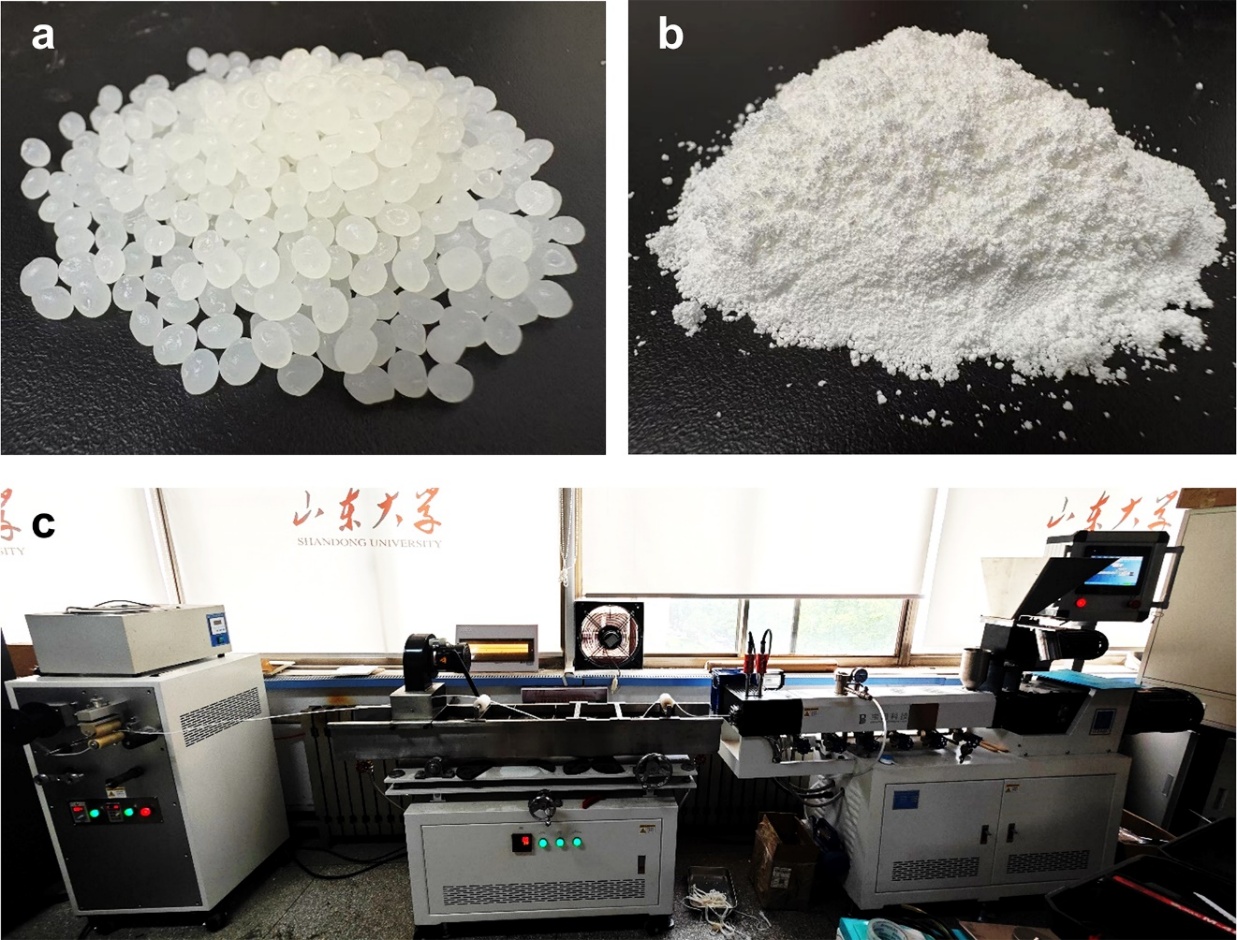
**

**Fig. S1** Illustration for continuous in-situ fibrillation methodology for processing PTFE/PLA composite fiber


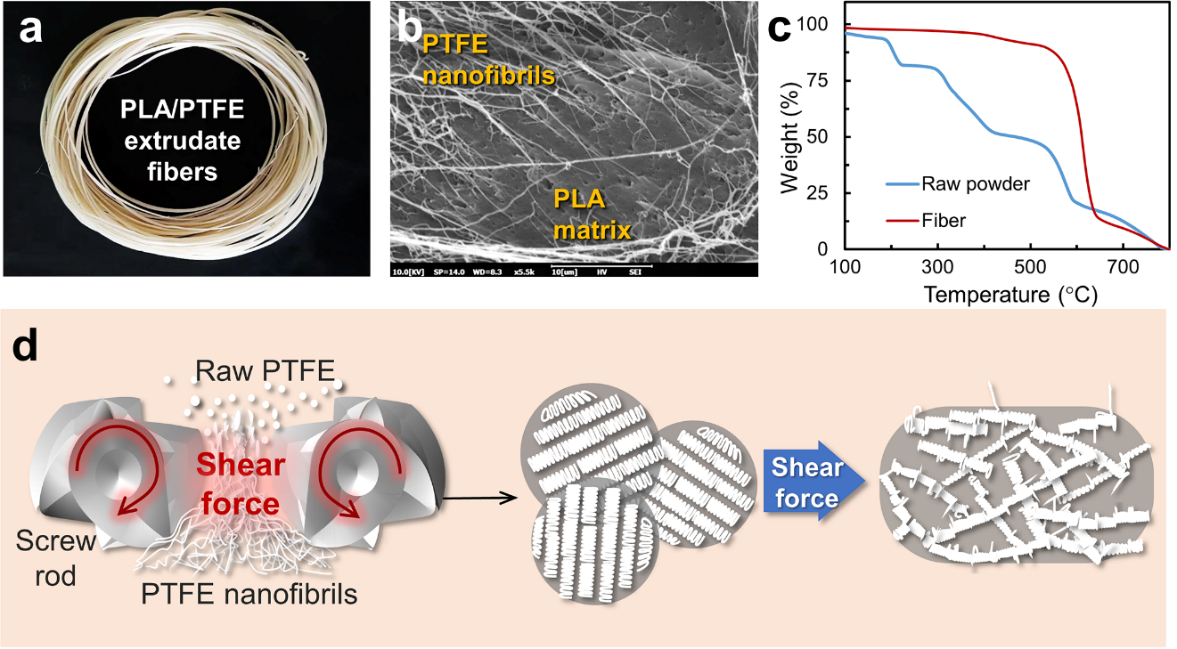


**Fig. S2** (**a**) Photograph of collected PLA/PTFE fibers. (**b**) SEM image of the fracture surface of PLA/PTFE fiber. (**c**) TGA curves for raw PTFE powder and obtained PTFE fibers. (**d**) Schematic illustration for the in-situ fibrillation mechanism


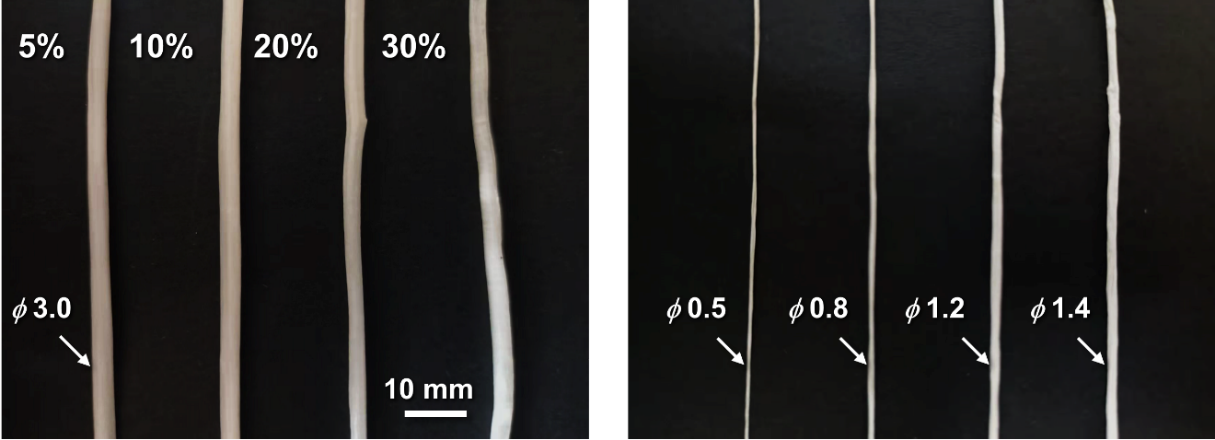


**Fig. S3** SEM images showing the PTFE/PLA composite fiber with varied weight ratio (left) and the obtained microporous PTFE fiber

**
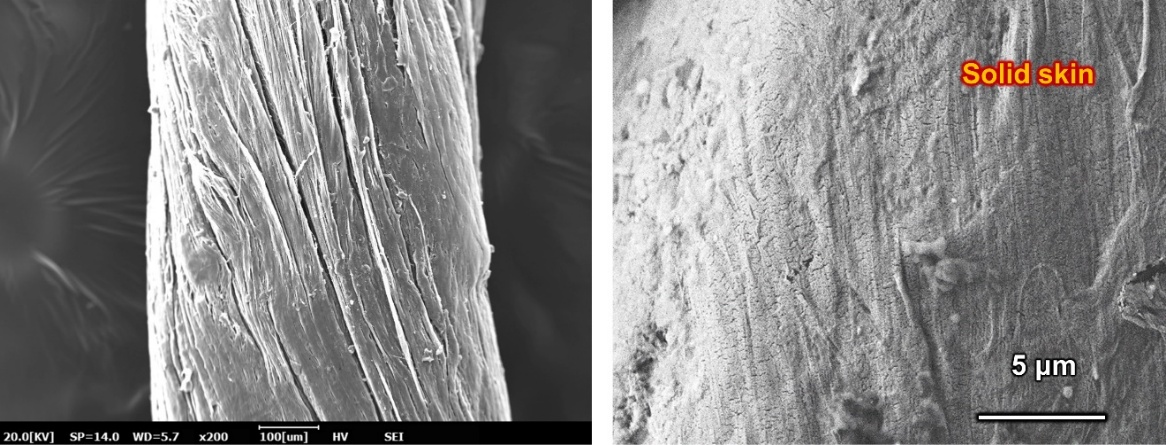
**

**Fig. S4** SEM images showing the dense skin layer of as-prepared microporous PTFE fiber

**
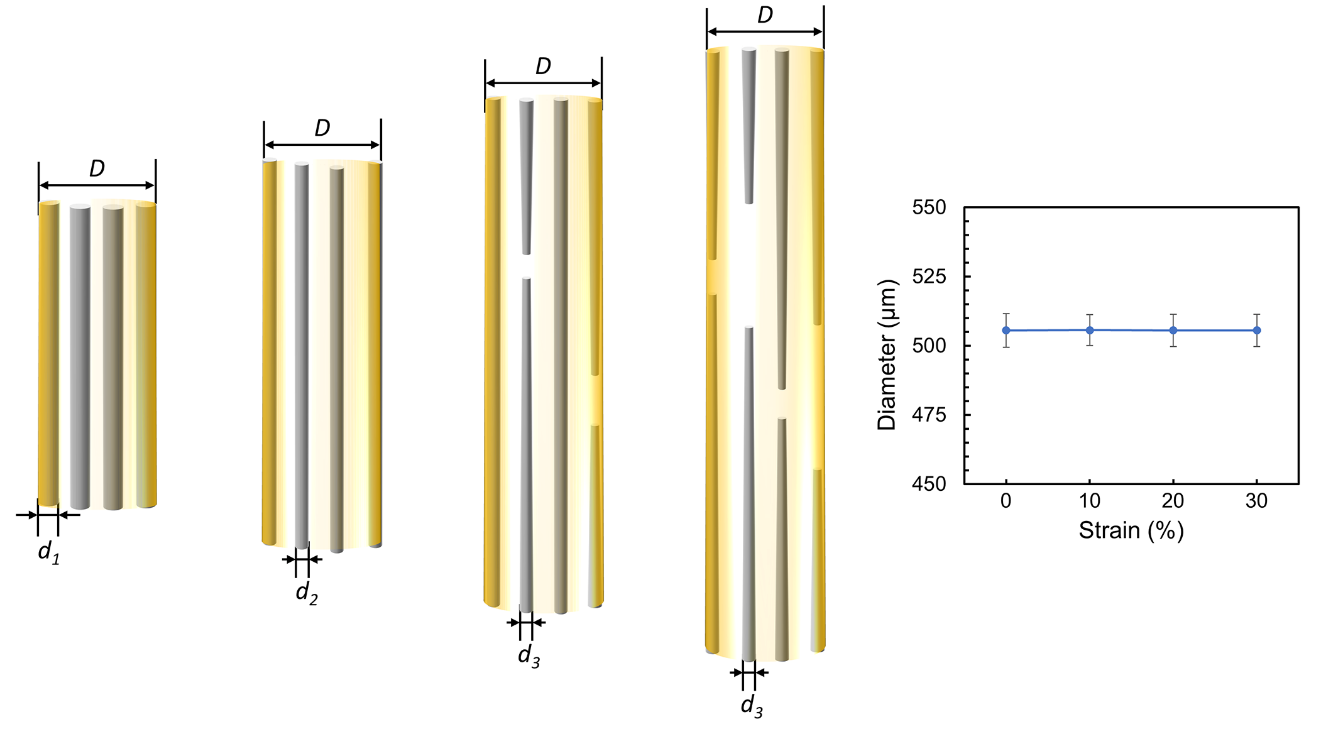
**

**Fig. S5** Schematic illustration of the zero Poisson’s ratio phenomenon (left) and the variation of fiber diameter with different tensile strain (right)


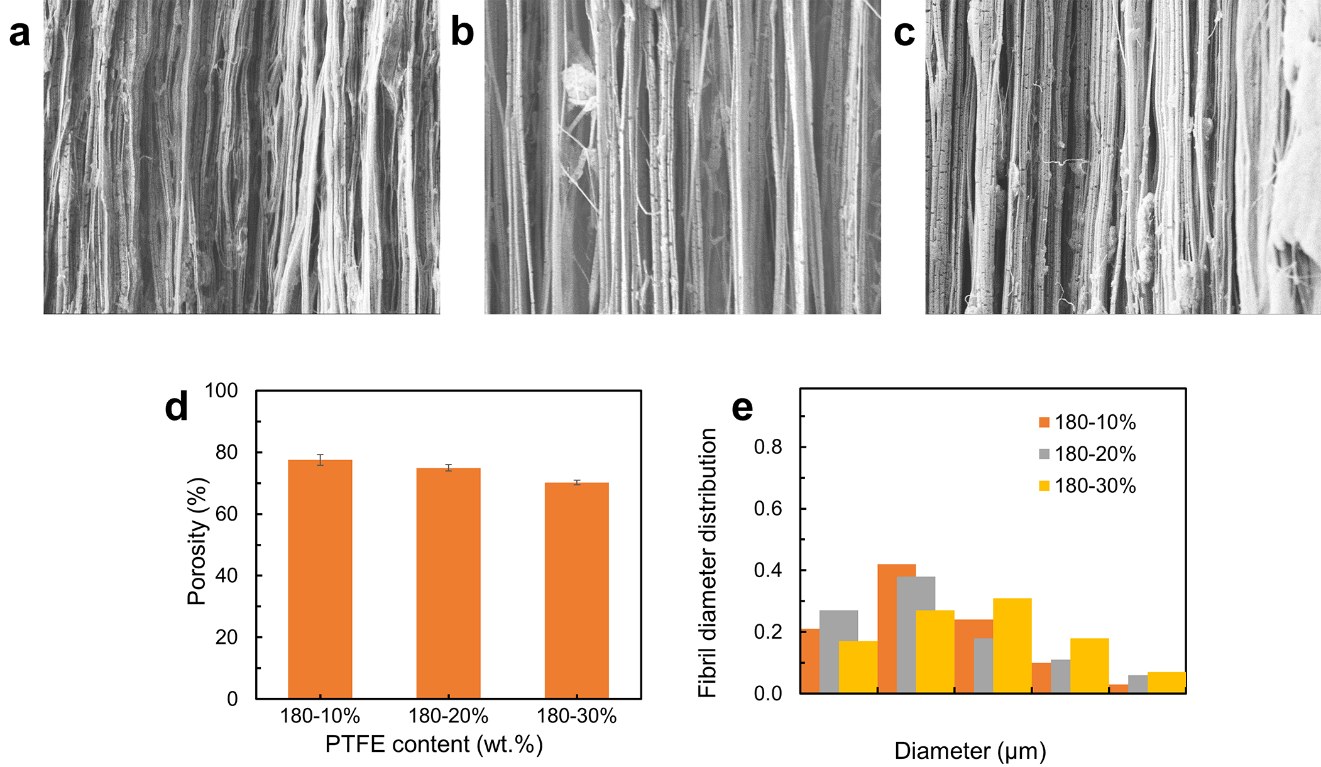


**Fig. S6** **Morphology of microporous PTFE fiber via different PTFE/PLA weight ratio.** SEM images of microporous PTFE fibers processed at different weight ratio of: **a** 10 wt.%, **b** 20 wt.%, and **c** 30 wt.%. **d** Porosity of microporous PTFE fibers with different PTFE/PLA weight ratio. **e** Diameter distribution of fibrils in PTFE fibers with different PTFE/PLA weight ratio
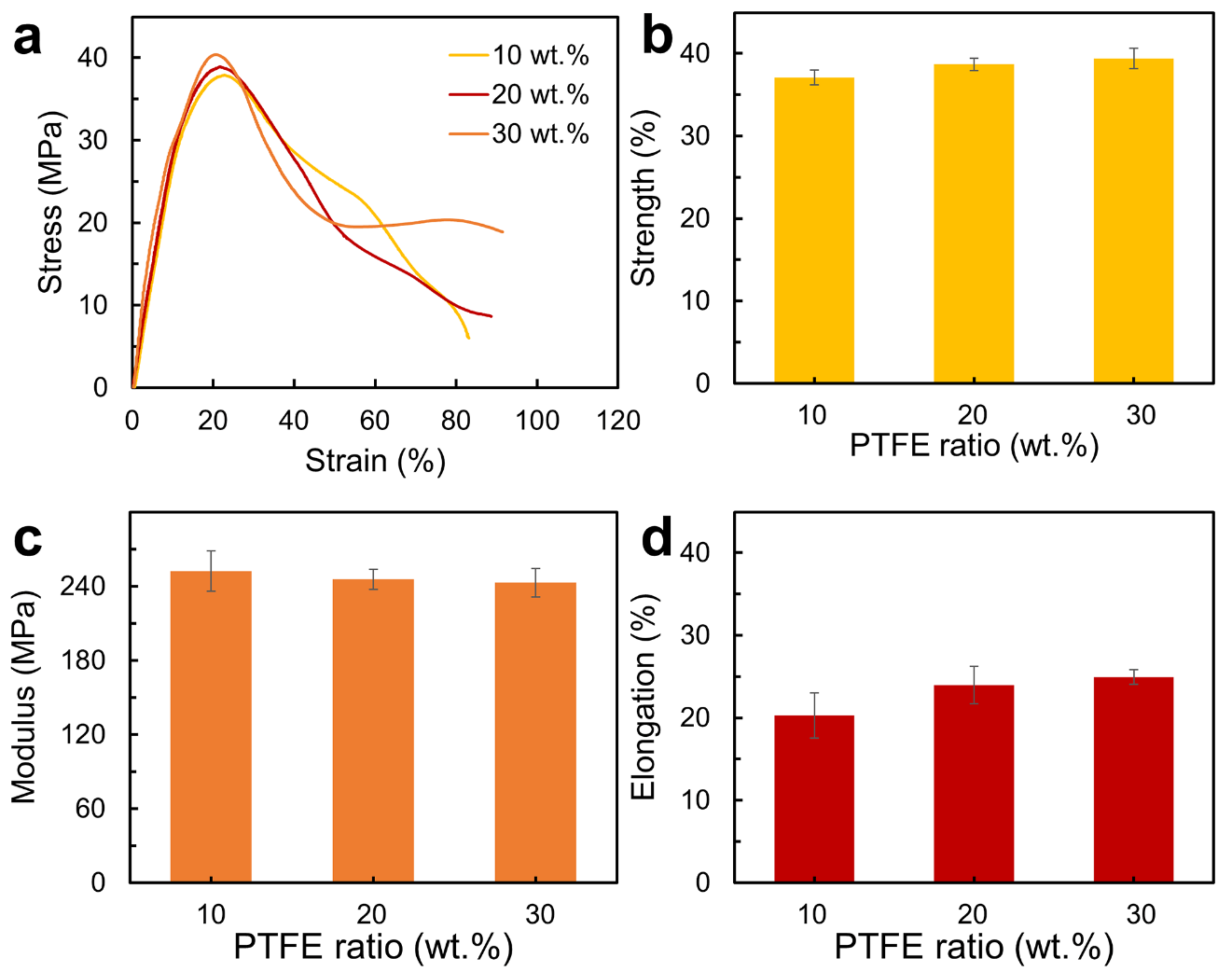


**Fig. S7** Mechanical properties of microporous PTFE fiber via different PTFE/PLA weight ratio. **a** Stress-strain curves. **b** Tensile strength. **c** Tensile modulus. **d** Elongation at break

**
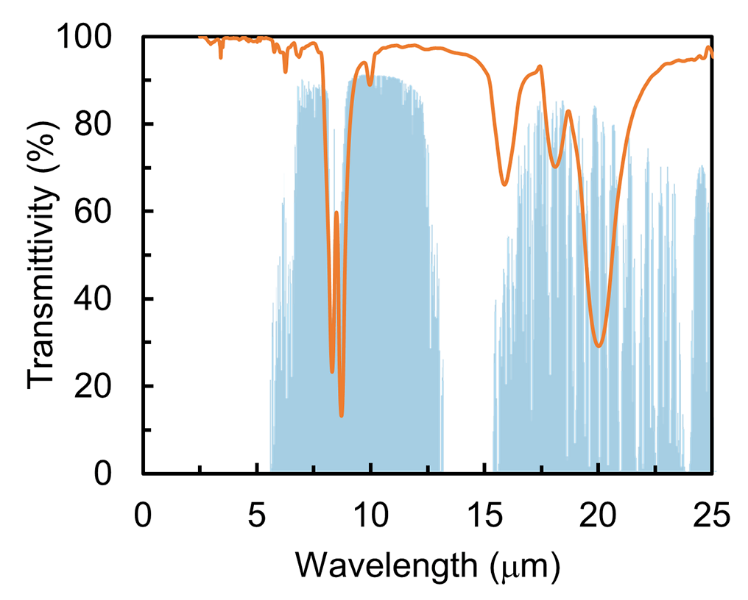
**

**Fig. S8** Infrared transmittance of as-prepared PTFE textile

**
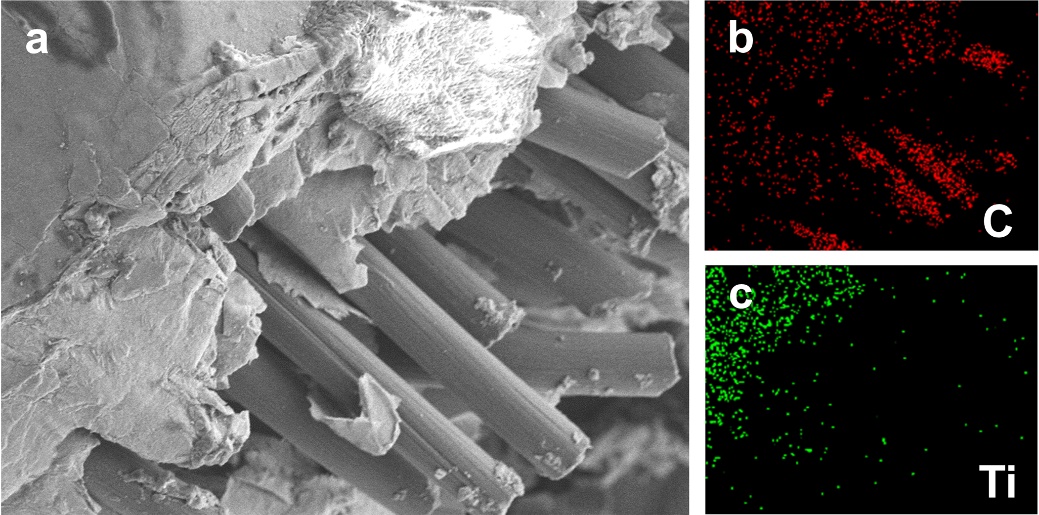
**

**Fig. S9** **Morphology of MXene coated carbon fabric. a** SEM image of the fabric. **b, c** Element mapping images of C and Ti elements


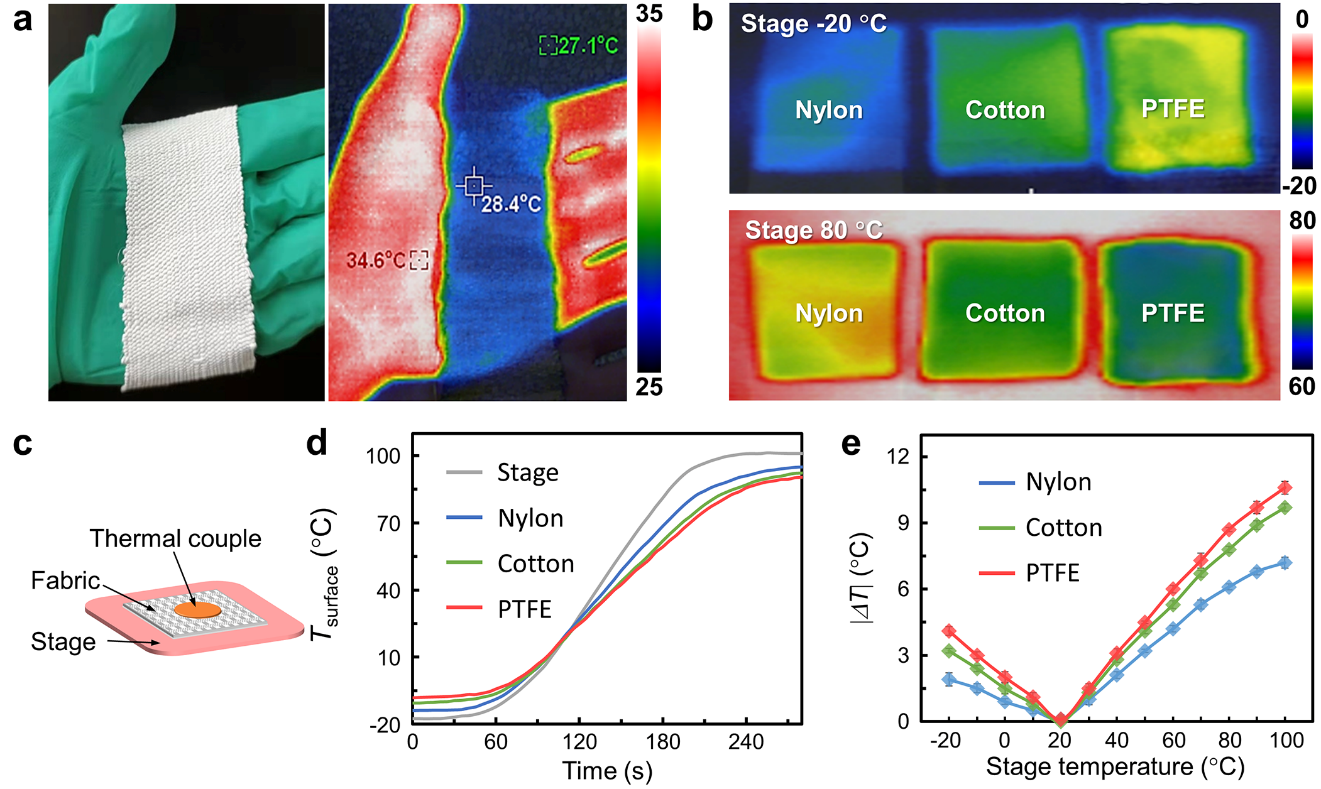


**Fig. S10** **Characterization of thermal insulation property of PTFE textile.** **a** Digital (left) and infrared (right) images of the PTFE textile. **b** Infrared images of PTFE, nylon, and cotton textiles placed on the cold stage (upper) and hot stage (bottom). **c** Schematic for the equipment for charactering thermal insulation property. **d** Temperature of the textile surface measured when changing the stage temperature from −20 to 80 °C. **e** Temperature difference (|Δ*T* |) between the textile surface and the stage against the stage temperature for different textiles


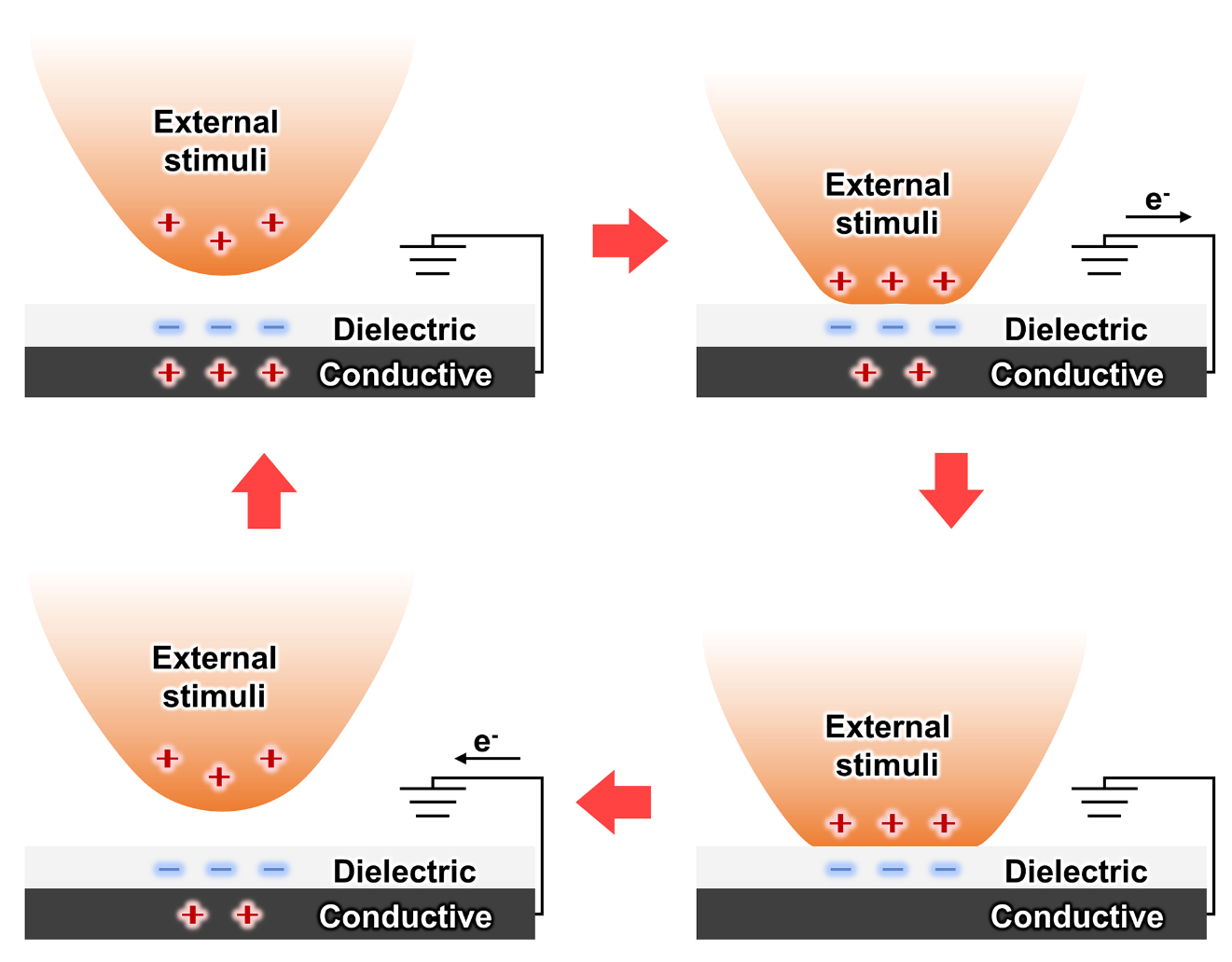


**Fig. S11** Schematic illustration of the working mechanism of X-Janus textile operating at single-electrode mode


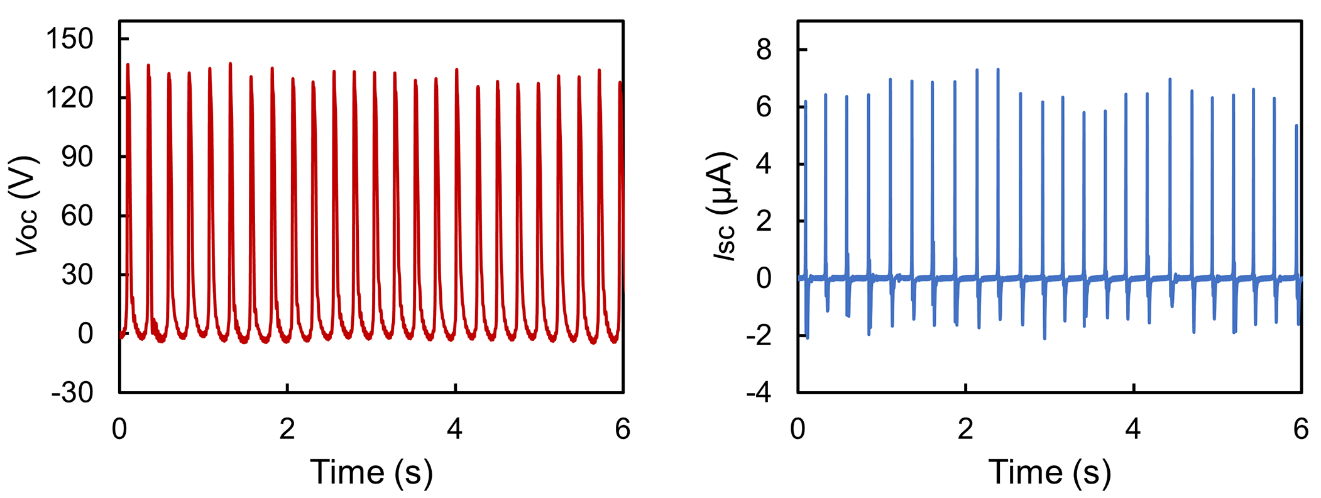


**Fig. S12** Electrical output of open-circuit voltage (left) and short-circuit current (right) using cotton fabric as the contact material


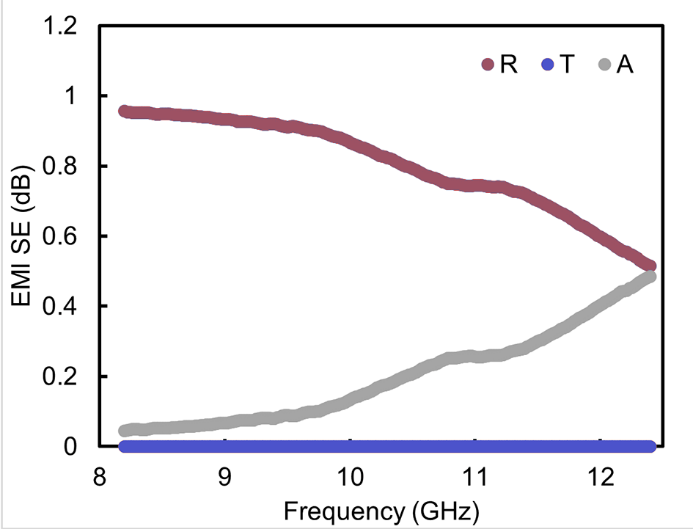


**Fig. S13** Reflection, absorption, and transmission coefficient of EMI SE effectiveness


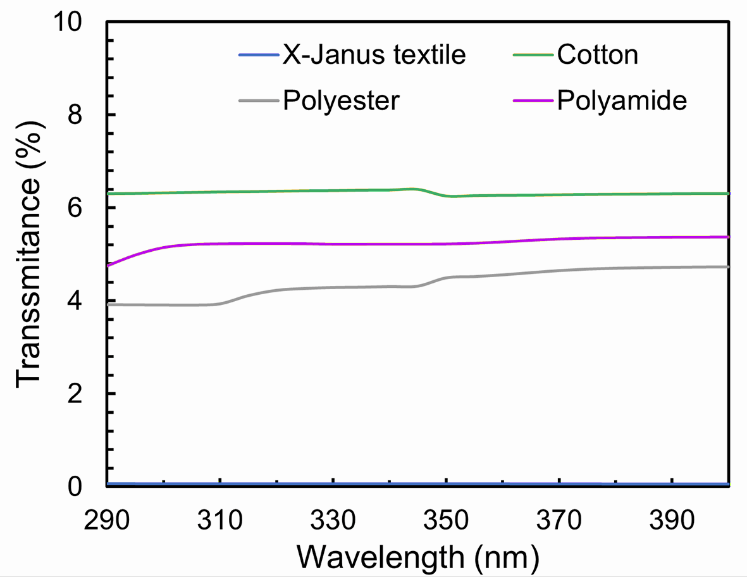


**Fig. S14** Comparison of UV-Vis-NIR transmittance of the X-Janus textile and other commercial textile

**Table S1.** Comparison of multifaceted Janus properties between this work and previously reported textiles.

| Refs. | Materials | Method | VL reflectivity | | IR emissivity | | Electrical conductivity | | Wettability | | Thermal conductivity | |
| --- | --- | --- | --- | --- | --- | --- | --- | --- | --- | --- | --- | --- |
|  |  |  | (%) | | (%) | | S/m | | Water contact angle (°) | | W·m^-1^·K^-1^ | |
| This work | PTFE+MXene/CF | In-situ fibrillation | 90 | 8 | 85 | 10 | ~10^-7^ | ~10^5^ | 0 | ~130 | 114 | 550 |
| [46] | PLA/ZnO+CB/Cu/PDMS@Ag/nylon+PE | Spinning+braiding | 90.0 | / | 90.5 | / | / | / | 0 | ~70 | / | / |
| [24] | PLA/ZnO+PU | Electro-spinning | 98.3 | / | 89.2 | / | / | / | / | / | / | / |
| [47] | CA+MXene | Electro-spinning | 96 | 11 | 88 | 14 | / | / | / | / | / | / |
| [48] | PDMS+CB | Water-regulated curing | 93 | 5 | 94 | / | / | / | / | / | / | / |
| [49] | PMMA/NaH2PO3+PPy/Cotton | Surface modification+blending | 92.6 | 2 | 97.2 | / | / | / | / | / | 89 | 112 |
| [26] | Carbon+Cu+Nano PE | Blade coating | ~90 | / | / | 20-60 | / | / | / | / | / | / |
| [50] | PU/Al_2_O_3_/TF & PU/CB/FA | Electro-spinning | 94.8 | 4.7 | 95 | 91.5 | / | / | / | / | / | / |
